# DNA replication fork speed underlies cell fate changes and promotes reprogramming

**DOI:** 10.1038/s41588-022-01023-0

**Published:** 2022-03-07

**Authors:** Tsunetoshi Nakatani, Jiangwei Lin, Fei Ji, Andreas Ettinger, Julien Pontabry, Mikiko Tokoro, Luis Altamirano-Pacheco, Jonathan Fiorentino, Elmir Mahammadov, Yu Hatano, Capucine Van Rechem, Damayanti Chakraborty, Elias R. Ruiz-Morales, Paola Y. Arguello Pascualli, Antonio Scialdone, Kazuo Yamagata, Johnathan R. Whetstine, Ruslan I. Sadreyev, Maria-Elena Torres-Padilla

**Affiliations:** 1 Institute of Epigenetics and Stem Cells, Helmholtz Zentrum München, München, Germany; 2 Department of Molecular Biology, Massachusetts General Hospital, Boston, MA USA; 3 Department of Genetics, Harvard Medical School, Boston, MA USA; 4 Faculty of Biology-Oriented Science and Technology, Kindai University, Wakayama, Japan; 5 Institute of Functional Epigenetics, Helmholtz Zentrum München, Neuherberg, Germany; 6 Institute of Computational Biology, Helmholtz Zentrum München, Neuherberg, Germany; 7 Massachusetts General Hospital Cancer Center and Department of Medicine, Harvard Medical School, Charlestown, MA USA; 8 Cancer Signaling and Epigenetics Program, Fox Chase Cancer Center, Philadelphia, PA USA; 9 Cancer Epigenetics Institute, Fox Chase Cancer Center, Philadelphia, PA USA; 10 Department of Pathology, Massachusetts General Hospital and Harvard Medical School, Boston, MA USA; 11 Faculty of Biology, Ludwig-Maximilians Universität, München, Germany

**Keywords:** Epigenetics, Genetics, Stem cells, Developmental biology

## Abstract

Totipotency emerges in early embryogenesis, but its molecular underpinnings remain poorly characterized. In the present study, we employed DNA fiber analysis to investigate how pluripotent stem cells are reprogrammed into totipotent-like 2-cell-like cells (2CLCs). We show that totipotent cells of the early mouse embryo have slow DNA replication fork speed and that 2CLCs recapitulate this feature, suggesting that fork speed underlies the transition to a totipotent-like state. 2CLCs emerge concomitant with DNA replication and display changes in replication timing (RT), particularly during the early S-phase. RT changes occur prior to 2CLC emergence, suggesting that RT may predispose to gene expression changes and consequent reprogramming of cell fate. Slowing down replication fork speed experimentally induces 2CLCs. In vivo, slowing fork speed improves the reprogramming efficiency of somatic cell nuclear transfer. Our data suggest that fork speed regulates cellular plasticity and that remodeling of replication features leads to changes in cell fate and reprogramming.

## Abstract (web summary)

Totipotent cells in mouse embryos and 2-cell-like cells have slow DNA replication fork speed. Perturbations that slow replication fork speed promote 2-cell-like cell emergence and improve somatic cell nuclear transfer reprogramming and formation of induced pluripotent stem cell colonies.

## Main

Cellular plasticity is an essential requirement for multicellular organisms. Cells in the early mammalian embryo are most plastic because they can generate every cell type in the body. In particular, the mouse zygote and each of the blastomeres in 2-cell-stage embryos are totipotent^1,2^, because they can generate a new organism on their own without the need for carrier cells. This contrasts with pluripotent cells, which can generate all the cells in the body, but not extraembryonic tissues^3,4^. Thus, totipotent cells have greater cellular plasticity. However, the mechanisms that sustain totipotency are poorly understood.

DNA replication is a fundamental process for genetic and epigenetic inheritance. However, how the early mammalian embryo replicates its DNA and whether the acquisition of totipotency is regulated through DNA-replication-dependent mechanisms is unknown. As the molecular properties of the replication fork are central to the regulation of replication^5^, we set out to investigate replication fork dynamics in totipotent cells in vivo and totipotent-like cells in culture.

## Results

### 2CLCs and totipotent embryos have a slow replication fork speed

**Fig. 1:**
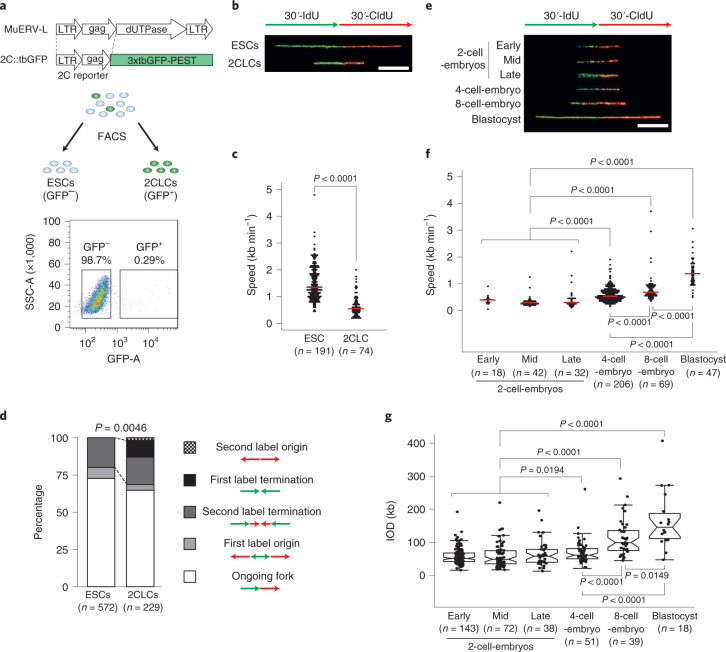
2CLCs and totipotent embryos display slow replication fork speed. **a**, Experimental setup for isolation of ESCs (GFP^−^) and 2CLCs (GFP^+^) based on FACS. **b**–**g**, DNA fiber analysis of pluripotent and totipotent cells by sequential labeling of nascent DNA. Representative fiber images (**b**) and quantification results of fork speed (**c**) from ESCs and 2CLCs are shown. **d**, Distribution of patterns of replication derived from fiber analyses from ESCs and 2CLCs. **e**,**f**, Representative fiber images (**e**) and quantification results of fork speed (**f**) from mouse embryos at the indicated developmental stages. **g**, Quantification of the IOD at the indicated stages of mouse preimplantation embryos. In **c** and **f**, the red line indicates the median. In **g**, the boxplots show the median and the IQR and whiskers depict the smallest and largest values within 1.5 × IQR. In **c**, **f** and **g**, statistical analyses were performed with a two-sided Wilcoxon’s rank-sum test. In **d**, statistical analyses were performed using a two-sided binomial test. In **b** and **e**, scale bars, 5 μm.

**Extended Data Fig. 1:**
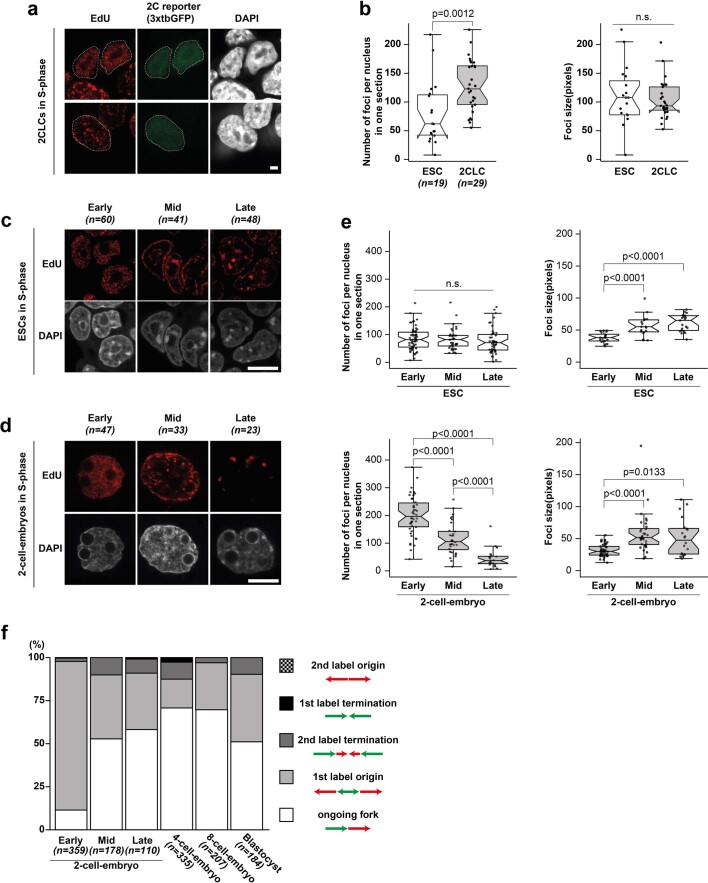
Increasing the number of active origins in 2-cell-embryos. **a**. DNA replication in asynchronous ESCs and 2CLCs visualized with STED microscopy. Cells were pulse-labeled with EdU for 20 min. White dotted line indicates 2CLCs. Scale bar, 2.5 μm. Images were acquired side by side and analyzed using identical parameters and are therefore comparable. **b**. Number and size distribution of EdU foci of representative, randomly selected ESCs and 2CLCs. Each dot indicates the number of EdU foci (left) and their size (right) in one single STED section in one nucleus. Boxplots: median and interquartile range (IQR); whiskers: smallest and largest values within 1.5×IQR. 1 pixel equals 20.6 nm. Statistical analyses: two-sided Wilcoxon rank-sum test. **c**. DNA replication patterns in ESCs under STED microscopy. EdU was added 0, 2, 4 h after double thymidine block release for early, mid, and late S-phase, respectively. **d**. DNA replication patterns in 2-cell-embryos using STED microscopy. EdU incubation was from 34, 36, and 38 h post-hCG injection for early, mid, and late S-phase, respectively. Images in c and d were acquired side by side, analyzed using identical parameters and are comparable (but not with panels in a and b). **c, d**, Scale bar, 10 μm; n, number of nuclei analyzed. **e**. Number and size distribution of replication foci of early, mid, and late S-phase in ESCs and 2-cell-embryos. Each dot indicates the number of EdU foci (left) and their size (right) in each nucleus in one STED section. Note that because the nuclear volume of 2-cell embryos is approximately 20 times bigger than ESCs, the total number of DNA foci in 2-cell embryos is much higher than ESCs. Boxplots: median and interquartile range (IQR); whiskers: smallest and largest values within 1.5×IQR. 1 pixel equals 20.6 nm. Statistical analyses: two-sided Wilcoxon rank-sum test. **f**. Replication patterns from fiber analyses at the indicated embryonic stages. N: number of fibers analyzed.

Totipotent-like cells resembling 2-cell-stage mouse embryos arise spontaneously in embryonic stem cell (ESC) cultures, but only in very low proportions of around 0.5%^6^. 2CLCs recapitulate several molecular features of the totipotent cells in mouse embryos and display expanded potency, including higher ability to be reprogrammed upon nuclear transfer^6–8^. Similar to 2-cell-stage embryos, 2CLCs express specific repeats such as MERVL^6,9^ and thus can be identified by a fluorescent reporter under the control of the MERVL long-terminal repeat^6,10^, enabling their characterization and isolation (Fig. 1a). We used DNA fiber analysis to study DNA replication and measure replication fork speed^11,12^. Analysis of replication fork speed in 2CLCs revealed a significantly slower fork speed compared with ESCs (Fig. 1b). Although ESCs displayed an expected rate of 1.34 kb min^−1^ (ref. ^13^), 2CLCs had approximately half this speed (0.56 kb min^−1^) (Fig. 1c). This suggested that totipotent-like cells in culture replicate DNA much more slowly than pluripotent stem cells. Importantly, the length of the S-phase did not change (see also below), suggesting that 2CLCs may use more origins than ESCs, to compensate for a slower fork progression. Indeed, analysis of the DNA fibers^14^ indicated an increase in DNA fibers in which replication stopped after the first label, implying more termination or blockage events (Fig. 1d), consistent with increased origin usage. In agreement, visualization of replication by 5-ethynyl-2′-deoxyuridine (EdU) incorporation revealed that 2CLCs displayed a more dispersed EdU pattern and higher number of replication clusters compared with ESCs (Extended Data Fig. 1a,b).

To address whether slow replication dynamics is a feature of genuine totipotent cells, we measured replication fork speed in 2-cell-stage embryos in vivo (Fig. 1e). Notably, 2-cell-stage embryos displayed a low fork speed during their complete S-phase (median 0.33 kb min^−1^ in early, mid and late S-phase; Fig. 1f). This was in contrast to 4- and 8-cell-stage embryos, which displayed faster replication dynamics that increased further at the blastocyst stage (0.53 kb min^−1^, 0.68 kb min^−1^ and 1.37 kb min^−1^, respectively; Fig. 1e,f). The slow fork speed in 2-cell-stage embryos was accompanied by an increase in the number of replication clusters compared with ESCs, considering the difference in nuclear volume (Extended Data Fig. 1c–e), suggestive of an increase in the number of replication foci and potentially also of origins used. To explore this possibility, we quantified the proportion of ‘origin label’ events as well as inter-origin distance (IOD). The 2-cell-stage embryos displayed a higher ratio of origins to forks (first label origin) on average confined to the early S-phase and compared with 8-cell-stage embryos and blastocysts (Extended Data Fig. 1f). In addition, the IODs—known to inversely correlate with the number of active origins^15^—were significantly shorter in 2-cell-stage embryos, compared with 8-cell embryos and blastocysts (Fig. 1g). Thus, totipotent cells in the early embryo replicate DNA with slow fork dynamics, which increases as development proceeds. These data underscore fundamental properties of DNA replication dynamics in the early mouse embryo.

### Emergence of 2CLCs requires DNA replication

**Fig. 2:**
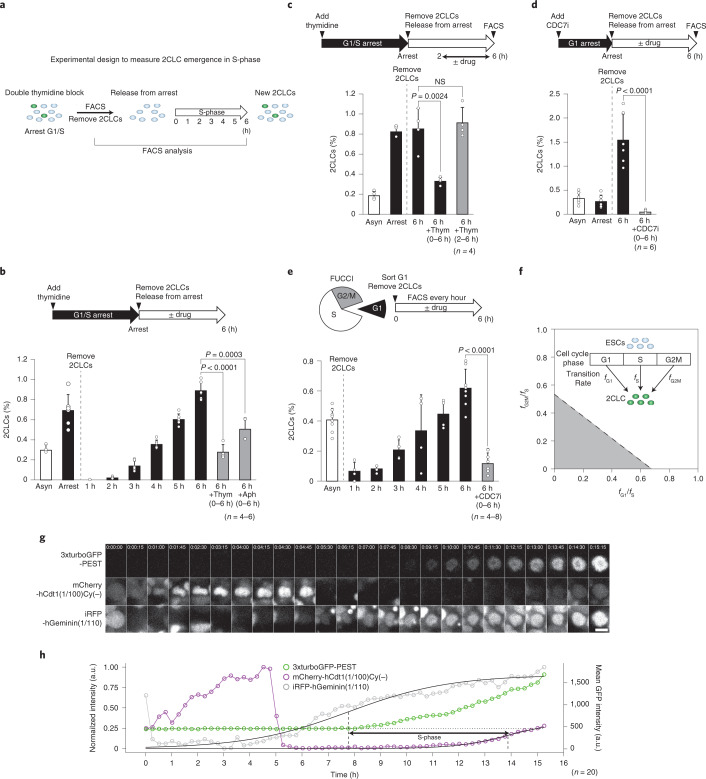
Emergence of 2CLCs occurs together with or after DNA replication. **a**, Strategy to evaluate 2CLC emergence during the S-phase. **b**, After synchronization of ESCs at G1/S by double thymidine block, existing 2CLCs were removed. The remaining cells were released from the block and cultured with or without the indicated inhibitors. Emerging 2CLCs were quantified by FACS. Asyn, asynchronized. **c**, After synchronization as in **b**, 2CLCs were removed by FACS and thymidine added 2–6 h after release to prevent S-phase completion. Emerging 2CLCs were quantified 6 h after release. NS, not significant. **d**, ESCs synchronized in G1 using a CDC7 inhibitor, after which existing 2CLCs removed. Cells were subsequently grown with or without CDC7 inhibitor and newly emerging 2CLCs were quantified 6 h after release. Barplots show mean ± s.d. Statistical analyses are by two-sided Student’s *t*-test. **e**, ESCs in G1 sorted based on their FUCCI (mCherry-hCdt1(1/100)Cy(−) and iRFP-hGeminin (1/110)) fluorescence and new 2CLCs quantified hourly by FACS. The means ± s.d. of at least four independent biological replicates are shown. Statistical analyses are by two-sided Student’s *t-*test. **f**. Mathematical modeling showing the quantitative relationships between the transition rates (*f*) of ESCs into 2CLCs during cell cycle phases (that is, *f*_G1_, *f*_S_ and *f*_G2M_). The transition rate is the probability that an ESC changes its fate to 2CLC during a given unit of time. The gray area demarcates all possible values compatible with the data: all the values of transition rates falling within the gray area fit the experimental data. As the dashed line cuts the *y* and *x* axes at values <1 for both G2/M over S (*f*_G2M_/*f*_S_, *y* axis) and G1 over S (*f*_G1_/*f*_S_, *x* axis), transitions from ESCs to 2CLCs must occur most frequently in the S-phase. **g**,**h**, Live-cell microscopy indicating that 2CLCs emerge concomitantly with S-phase progression. a.u., arbitrary units. Live-imaging stills representative of 20 time-lapse recordings of emerging 2CLCs using FUCCI (mCherry-hCdt1(1/100)Cy(−) and iRFP-hGeminin (1/110)). **h**, Quantification of the representative emerging 2CLC in **g** depicting normalized mean fluorescence intensities (mCherry, iRFP, left axis) and mean raw fluorescence (GFP, right axis) over time. The S-phase duration is indicated. The majority of cells analyzed displayed similar results, with onset of 2C::tbGFP fluorescence during the S-phase or S/G2 transition. Scale bar, 10 μm. Barplots, mean ± s.d.; dots, values of each replicate; *n*, number of biological replicates.

**Extended Data Fig. 2:**
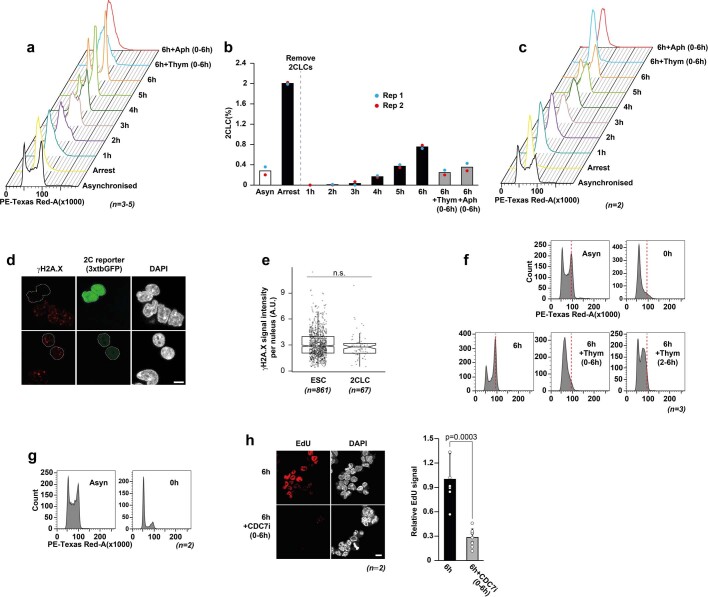
Effect of cell cycle progression on the emergence of 2 CLCs. **a**. Cell cycle profiles determined by FACS based on propidium iodide staining of ESCs after release from double thymidine block, which corresponds to Fig. 2b. **b**. Population of 2CLCs (in %) after release from double thymidine block detected with 2C::tdTomato reporter^6^. Following synchronization of ESCs at G1/S by double thymidine block, existing 2CLCs were removed and the remaining cells were released from the block and cultured with or without the indicated DNA replication inhibitor. Newly emerging 2CLCs were quantified by FACS at indicated time points after release. **c**. Cell cycle profiles determined by FACS based on propidium iodide staining of the 2C::tdTomato ESCs reporter line after release from double thymidine block, which corresponds to Extended Data Fig. 2b. **d, e**. γH2A.X immunostaining in asynchronous ESCs and 2CLCs. Representative images (d) and the corresponding quantification of global γH2A.X levels (e). 2CLCs are outlined with white dotted lines. Boxplots show median and interquartile range (IQR), whiskers depict the smallest and largest values within 1.5×IQR. Scale bar, 10 μm. Statistical analyzes: two-sided Wilcoxon rank-sum test. **f**. Cell cycle profiles determined by FACS based on propidium iodide staining of ESCs under thymidine treatment from 2 h after release from double thymidine block, which corresponds to Fig. 2c. The dashed, red line indicates the G2/M peak. **g, h**. S-phase progression after removal of the CDC7 inhibitor. The effects of CDC7 inhibitor on the cell cycle arrest (g) and restoration of the DNA replication after releasing from block (h, left and right panels) were verified by FACS and EdU incorporation, respectively. In **b** mean values are shown, dots indicate values for each replicate. In **h**, bar plots show mean±S.D and dots indicate values of each image. n indicates number of independent biological replicates. Statistical comparisons: two-sided Student’s *t*-test.

**Extended Data Fig. 3:**
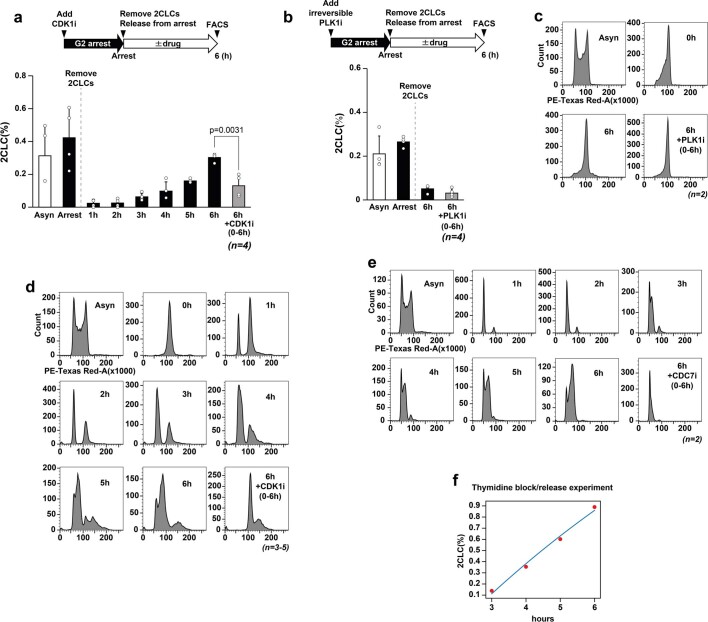
Effect of entry into S-phase on 2CLC reprogramming. **a**. ESCs were synchronized using a CDK1 inhibitor, after which existing 2CLCs were removed. Cells were subsequently grown with or without CDK1 inhibitor and newly emerging 2CLCs were quantified hourly by FACS until 6 h after block release. **b**. ESCs were synchronized using a PLK1 inhibitor, after which existing 2CLCs were removed. Cells were subsequently grown with or without PLK1 inhibitor and newly emerging 2CLCs were quantified by FACS 6 h after block release. **c**. Cell cycle profiles determined by FACS for propidium iodide staining of ESCs after release from PLK1 inhibitor, which corresponds to Extended Data Fig. 2b. **d**. Cell cycle profiles based on propidium iodide content of ESCs after release from CDK1 inhibitor, which corresponds to Extended Data Fig. 2a. **e**. Cell cycle profiles based on propidium iodide content of ESCs after collection in G1-phase using FACS without the addition of drugs (for example without cell cycle synchronization) based on the FUCCI reporter. These data correspond to Fig. 2e. **f**. The panel shows the data from the double thymidine block and release experiment described in Fig. 2A and the corresponding fit. The estimation of *f*_*s*_ obtained from this fit was then used to identify the values of $\frac{{f_{G1}}}{{f_S}}$ and $\frac{{f_{G2M}}}{{f_S}}$ compatible with the data, shown in Fig. 2f. These values lie on a line, shown in Fig. 2f, computed from eq. (1) in the Methods. The values of the parameters used are: $\frac{{N_{2c}}}{{N_E}} = 0.01,T_c = 8\;h,$ $\frac{1}{{\omega - \varphi _{2c}}} = 12\;h.$ In **a** and **b**, the bar plots show mean±S.D. and dots indicate the values of each replicate. n indicates the number of independent biological replicates. Statistical comparisons were performed by two-sided Student’s *t*-test.

Next, we reasoned that, if replication fork dynamics is relevant for 2CLC reprogramming, the S-phase may play a critical role for 2CLC emergence. To address this, we synchronized cells at G1/S using a double thymidine block, after which we removed pre-existing 2CLCs from the culture using fluorescence-activated cell sorting (FACS), and measured the number of newly emerging 2CLCs every hour after releasing the culture from the block, using the 2C MERVL-driven reporter as readout (Fig. 2a). This analysis revealed 2CLC emergence along with cell cycle progression, which reached the same proportion as the synchronized population on completion of the S-phase within ~6 h after release (Fig. 2b and Extended Data Fig. 2a). Inhibition of DNA synthesis, upon addition of aphidicolin or thymidine after release from G1/S, led to a reduction in the proportion of 2CLCs. This suggests that, although DNA synthesis partially contributes to 2CLC emergence, it is entry into the S-phase, which neither thymidine nor aphidicolin blocks, that is important for 2CLC reprogramming (Fig. 2b). We obtained similar results using another ESC line^6^ (Extended Data Fig. 2b,c). We also investigated whether 2CLC induction is related to checkpoint activation, but obtained no evidence for the requirement of checkpoint activation in 2CLC induction or increased γH2A.X levels in 2CLCs compared with ESCs^10^ (Extended Data Fig. 2d,e). To address whether completion of the S-phase is important for 2CLC induction, we added thymidine 2 h after release from the G1/S block. This resulted in full 2CLC induction (Fig. 2c), in concordance with cells progressing through the S-phase but accumulating before the G2/M peak (Extended Data Fig. 2f), suggesting that entry into the S-phase, but not necessarily completion, is relevant for 2CLC reprogramming. We then asked whether preventing origin firing affects 2CLC emergence. G1 synchronization and sustained treatment with a CDC7 kinase inhibitor (Extended Data Fig. 2g,h), which blocks MCM phosphorylation and thereby origin firing^16^, resulted in an almost complete suppression of 2CLC emergence (Fig. 2d). Importantly, synchronization at the G2/M transition did not increase the proportion of 2CLCs (Extended Data Fig. 3a), suggesting that our results do not reflect cell cycle inhibition in general, but rather reflect 2CLC emergence together with DNA replication. In agreement, irreversible cell cycle arrest prevented 2CLC emergence (Extended Data Fig. 3b,c). Of note, we observed an increase in 2CLCs after G2/M release, which paralleled progression into the next S-phase and was prevented on CDK1 inhibition, which blocks origin firing^17^ (Extended Data Fig. 3a,d). As cell cycle arrest using chemical inhibitors may have indirect effects, we looked at whether 2CLCs emerge during the S-phase in normal, cycling ESCs. Sorting G1 cells using the FUCCI (fluorescence ubiquitination cell cycle indicator) system^18^ in the absence of any chemical arrest confirmed de novo 2CLC emergence coincident with S-phase progression (Fig. 2e and Extended Data Fig. 3e). We also performed mathematical modeling using our cell cycle data (Extended Data Fig. 3f and Methods), which indicated that 2CLCs emerge primarily during the S-phase, because the transition rates (*f*) in other phases of the cell cycle are negligible and smaller than the transition rate in the S-phase (that is, *f*_G1_, *f*_G2M_ < *f*_S_; Fig. 2f). Accordingly, direct observation with live microscopy using the FUCCI system indicated that most 2CLCs emerge together with S-phase progression (Fig. 2g,h). We conclude that 2CLC emergence occurs concomitant with DNA replication and that entry into the S-phase is key for this reprogramming.

### Slowing replication fork speed induces 2CLCs

**Fig. 3:**
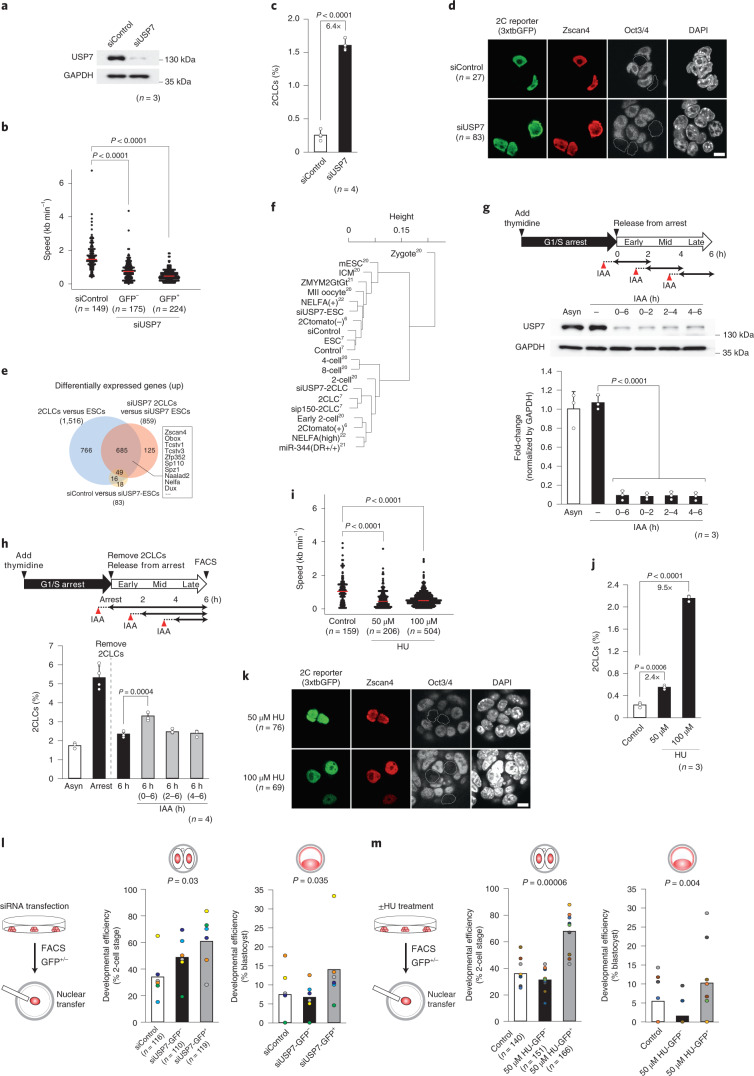
Slowing replication fork speed induces 2CLCs. **a**, USP7 expression 48 h after siRNA transfection. **b**, Fork speed in ESCs, GFP^+^ (Usp7KD-induced 2CLCs) and GFP^−^ cells after USP7 depletion. Statistical analysis was by two-sided Wilcoxon’s rank-sum test. **c**, FACS quantification of 2CLCs 48 h after USP7 siRNA transfection. Statistical analysis was by two-sided Student’s *t*-test. **d**, ZSCAN4 and OCT4 immunofluorescence in 2CLCs induced upon USP7 knockdown. **e**, Venn diagram of upregulated genes in control, USP7-depleted ESCs and USP7-depleted 2CLCs. **f**, Dendrogram of transcriptomes from various 2CLCs, early embryos, siControl-transfected ESCs, siUSP7-transfected ESCs and siUSP7-transfected 2CLCs. **g**,**h**, Early S-phase is critical for 2CLC induction on USP7 depletion. **g**, Western blot in an AID–USP7 knock-in cell line at indicated hours of auxin (indole-3-acetic acid (IAA)) treatment. IAA was added 30 min before early, mid or late S-phase (red arrowhead). GAPDH, glyceraldehyde 3-phosphate dehydrogenase. **h**, ESCs synchronized with double thymidine block, existing 2CLCs removed by FACS and IAA added as indicated. Emerging 2CLCs were quantified 6 h after release. Statistical analyses for pairwise comparison with control group were with a two-sided Student’s *t*-test. **i**, Fork speed in HU-treated ESCs. Statistical analyses were by Wilcoxon’s rank-sum test. **j**, 2CLCs induced by HU. The apparent higher increase in 2CLC percentage in 100 µM HU compared with 50 µM HU may be due to selective increase in ESC death and an increase in the number of cells in the S-phase with 100 µM HU (Extended Data Fig. 7j). Statistical analyses for pairwise comparison with control group used a two-sided Student’s *t*-test. **k**, ZSCAN4 and OCT4 immunofluorescence in 2CLCs induced by HU. **l**,**m**, Greater reprogrammability of 2CLCs, induced by slowing fork speed. Nuclei of sorted GFP^+^ and GFP^−^ cells after USP7 siRNA (**l**) or HU (**m**) treatment were transferred into enucleated oocytes. Reprogramming efficiency is indicated by development of NT-derived embryos to 2-cell (left) and blastocyst (right). Barplots show average percentage of developmental efficiency across 6 (**l**) and 10 (**m**) independent experiments; each dot indicates percentages obtained in each experiment and color depicts side-by-side experiments; *n*, number of embryos analyzed. Statistical analyses were by two-sided Welch’s test for unequal variances. **b**,**i**, Red line: median; barplots: mean ± s.d.; dots, values of each replicate; *n*, number of independent biological replicates. In **d** and **k**, scale bars, 10 μm. Source data

**Extended Data Fig. 4:**
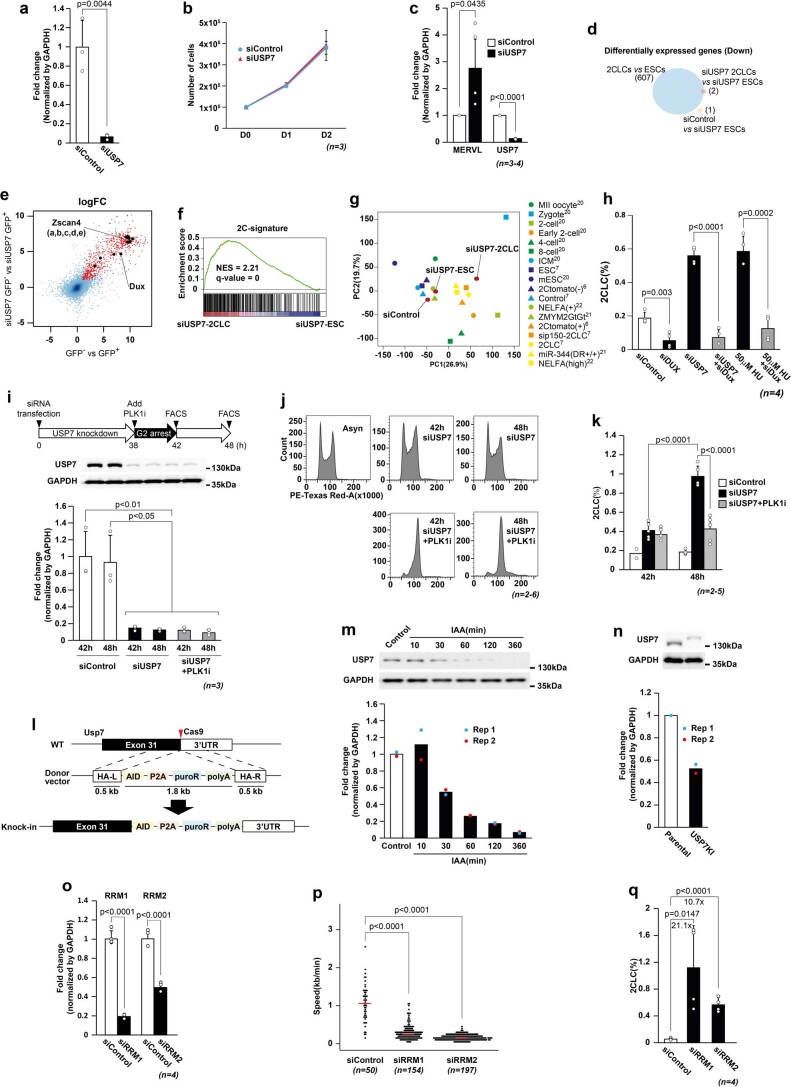
S-phase-dependent effect of USP7 on the emergence of 2CLCs. **a**. USP7 protein quantification upon *Usp7* siRNA (corresponds to Fig.3a). Statistical comparisons: two-sided Welch’s *t*-test. **b**. Growth curve after *Usp7* RNAi at day 1(D1) 2(D2) after seeding(D0). **c**. MERVL and *Usp7* qRT-QPCR upon *Usp7* RNAi. Statistical comparisons: two-sided Welch’s *t*-test. **d**. Venn diagram comparing downregulated genes in control, USP7-depleted ESCs, and USP7-depleted 2CLCs. **e**. logFC scatter plots between siUSP7-2CLCs and endogenous 2CLCs showing high overlap of upregulated genes (red) between both 2CLC samples. **f**. Gene-set enrichment analysis of siUSP7-induced 2CLCs against a ‘2C’ signature. **g**. PCA of siControl, siUSP7 ESCs (GFP^−^ cells) and siUSP7-induced 2CLCs transcriptomes compared with embryos and other 2CLC datasets. **h**. 2CLC percentage upon siRNA for *Usp7* or HU treatment combined with siRNA for *Dux*. Statistical comparisons: two-sided Student’s *t*-test for pairwise comparison only between the indicated groups. **i**. Western Blot after *Usp7* siRNA or control siRNA transfection and treatment of PLK1 inhibitor at indicated times. Statistical comparisons: two-sided Student’s *t*-test between each sample. Highest p-values are shown. **j-k**. Cell cycle profiles of ESCs (j) and 2CLC percentage (k) after transfection of control or *Usp7* siRNA, followed by treatment with PLK1 inhibitor at indicated times. In (k) n for 42 h control samples is 2 biological replicates; for 48 h control is 4 and for all other samples is 5. Statistical comparisons between the indicated groups: two-sided Student’s *t*-test. **l**. *Usp7* gene locus and knock-in strategy to insert Auxin-Inducible Degron (AID) at the C-terminus of USP7. **m**. Western Blot of USP7 after auxin (IAA) treatment. **n**. Western Blot in parental and knock-in USP7-AID line. The USP7-AID transgene causes lower USP7 expression compared to the parental clone, presumably leading to higher steady-state 2CLC population. **o**. *Rrm1* and *Rrm2* RT-qPCR 48 h after transfection with their respective siRNAs compared to control. Statistical comparisons: two-sided Welch’s *t*-test. **p**. Fork speed upon RNAi for RRM1, RRM2 or control. Statistical analysis: two-sided Wilcoxon rank-sum test. **q**. 2CLCs quantification 48 h after siRNA transfection. Statistical analysis: two-sided Student’s *t*-test. In bar graphs plots are mean±S.D and dots are individual replicate values. n: number of independent biological replicates. In m and n, dots are values from biological replicates.

To address how S-phase enables 2CLC reprogramming and given our observations above (Fig. 1), we focused on replication fork speed. We asked whether modulating replication fork speed can regulate reprogramming toward 2CLCs. For this, we sought to reduce fork speed experimentally. The USP7 deubiquitinase modulates small ubiquitin-like modifier (SUMO) levels at sites of DNA replication, thereby regulating replication fork progression. Inhibiting USP7 decreases fork speed in human cells and fibroblasts^19^. We thus asked whether ubiquitin-specific-processing protease 7 (USP7) depletion can induce 2CLCs. *Usp7* downregulation in ESCs led to reduced fork speed (Fig. 3a,b and Extended Data Fig. 4a) without significantly affecting cell proliferation (Extended Data Fig. 4b). Strikingly, *Usp7* RNA interference (RNAi) led to more than approximately sixfold induction of 2CLCs (Fig. 3c) and a concomitant increase in the transcription of the MERVL retrotransposon (Extended Data Fig. 4c), a marker of 2CLCs and 2-cell-stage embryos. The 2CLCs induced upon *Usp7* knockdown displayed typical 2CLC features, such as ZSCAN4 expression, downregulation of OCT4 (POU5F1), chromocenter dispersion (Fig. 3d) and a high gene expression profile overlap with endogenous 2CLCs^7^ (Fig. 3e and Extended Data Fig. 4d), including upregulation of MERVL and MT2_Mm and an enrichment of ‘2C’ genes (Supplementary Tables 1 and 2 and Extended Data Fig. 4e–g). Unsupervised clustering of transcriptomes from early embryos^20^, ESCs and several 2CLC datasets^6,7,21,22^ confirmed that USP7 knockdown-induced 2CLCs are transcriptionally more similar to 2CLCs and 2-cell-stage embryos (Fig. 3f). In line with their 2CLC identity^8,23^, they express the transcription factor *Dux* and MERVL activation—as determined using the 2C reporter—that was dependent on *Dux* (Fig. 3e and Extended Data Fig. 4h). As USP7 can have multiple functions throughout the cell cycle^24,25^, we next asked whether USP7 functions to regulate 2CLC emergence during or outside the S-phase. For this, we first depleted USP7 using small interfering (si)RNA, then synchronized cells at the G2/M transition using a PLK1 (polo-like kinase 1) inhibitor (PLKi) and cultured them for another 6 h (Extended Data Fig. 4i,j), after which we determined the number of 2CLCs. Addition of the PLK1i prevented induction of 2CLCs after synchronization at G2/M (Extended Data Fig. 4k), suggesting that the effect of USP7 depletion in inducing 2CLCs occurs before G2. To address this directly, we engineered a knock-in ESC homozygous *Usp7* allele with an auxin-induced degron (AID) (Extended Data Fig. 4l), which enables precise temporal control of USP7 protein using auxin (Extended Data Fig. 4m). With this approach, we were able to deplete USP7 specifically from the early, mid or late S-phase (Fig. 3g). Using these conditions, we determined the impact of the temporal depletion of USP7 on 2CLC emergence in the S-phase immediately after release from double thymidine block as above (Fig. 3h). The steady-state population of 2CLCs was higher in the USP7–AID cell line, presumably because our transgene causes slightly lower USP7 expression compared with the parental clone (Extended Data Fig. 4n). USP7 depletion resulted in a 2CLC increase, compared with basal levels, exclusively when depleted from early S-phase onward, but not from mid or late S-phase (Fig. 3h). These experiments demonstrate that entry into the S-phase and/or early S-phase is critical for 2CLC emergence.

**Extended Data Fig. 5:**
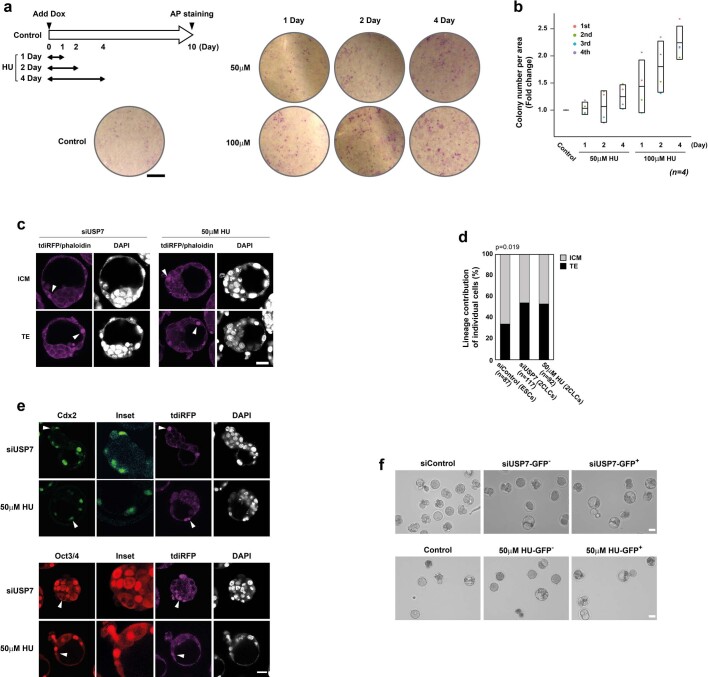
Impact of modulating replication fork speed on reprogramming. **a, b**. HU treatment facilitates iPS reprogramming. Alkaline phosphatase-positive iPSC colonies (a) and their quantification results (b) after OKSM induction by Dox in reprogrammable MEFs treated during the indicated time windows of HU. In **b**, the dots indicate the values from individual experiments compared to the control, the middle line is the mean and the boxes depict mean ± SD. Statistical analyses were performed with a generalized linear model using a Poisson distribution. Both the concentration and the days as well as the combination have a significant effect (p < 0.0001) on reprogramming efficiency. Scale bar, 1 mm. **c**. **d**. Lineage contribution of ESCs and 2CLCs in chimeric blastocysts. siControl-transfected ESCs, siUSP7-induced 2CLCs, and HU-induced 2CLCs were aggregated with 4–8 cell stage embryos and cultured for 2 days. Blastocysts were analyzed by confocal microscopy, and reconstructed in 3D to determine the position of individual cells in each lineage using phalloidin as cell membrane label. Representative images of cells within ICM and TE (c) and the quantification results (d) of 26, 38 and 32 embryos analyzed per group, respectively, are shown. Data are displayed as the percentage of cells, which upon aggregation, display inner (ICM) or outer (TE) position. Statistical analyses were performed with a Kruskal-Wallis test. Scale bar, 25 μm. **e**. Representative images showing immunostaining of chimeric blastocysts injected with H2B-tdiRFP expressing siUSP7-2CLCs or 50 μM HU induced 2CLCs. H2B-tdiRFP-positive cells expressing Cdx2 and Oct3/4 are indicated by arrowheads and the corresponding Insets at higher magnification are shown. A total of 17 embryos were injected with siUSP7-2CLCs and 21 embryos were injected with HU induced 2CLCs. Note that the Oct3/4 positive cell in the siUSP7 panel depicts a cell in mitosis. Scale bars, 25 μm. **f**. Representative images of nuclear transferred embryos derived from the indicated donor cells 4 days after activation, corresponding to a representative experiment related to Figs. 3l and m. Scale bar, 50 μm.

As an orthologous approach to slow down replication fork speed, we employed low doses of hydroxyurea (HU)^26^. We verified that HU treatment led to a reduction in fork speed (Fig. 3i). HU treatment resulted in a striking, approximately tenfold increase in 2CLCs (Fig. 3j), which displayed typical 2CLC features (Fig. 3k and Extended Data Fig. 4h). As a third approach, we used RNAi to achieve partial downregulation for the ribonucleotide reductase subunits RRM1 and RRM2, known to result in reduction of fork speed^26,27^(Extended Data Fig. 4o,p). Downregulation of both RRM1 and RRM2 led to a robust 2CLC increase of ~20- and 10-fold, respectively (Extended Data Fig. 4q), suggesting that slowing the replication fork speed regulates changes in cell fate and highlighting the relevance of replication fork dynamics for 2CLC reprogramming. We also addressed whether our findings may be applicable to other reprogramming systems. Namely, we addressed whether induced pluripotent cell (iPSC) generation can be improved upon incubation with low doses of HU. Our results (Extended Data Fig. 5a,b) indicate an increase in the number of iPSC colonies after exposure to HU and may suggest a more general role for fork speed in cell reprogramming.

To further characterize the 2CLCs induced by USP7 depletion or HU, we examined their developmental potential compared with ESCs using two approaches. First, we performed morula aggregation with ESCs and 2CLCs produced after USP7 downregulation or by HU treatment, and analyzed their lineage contribution in blastocysts reconstructed in three dimensions, based on confocal microscopy. In each experiment we aggregated an equivalent number of cells and scored the number of cells in the inner cell mass (ICM) or the trophectoderm (TE) to account for variability between embryos. Although we found ESCs contributing to both the ICM and the TE, with a strong bias toward the ICM, in agreement with previous reports under these conditions^28–30^, 2CLCs more frequently contributed to both (Extended Data Fig. 5c,d), in line with the suggested bipotentiality of 2CLCs. Single-cell chimera injections confirmed that 2CLCs can contribute to cells that express OCT4 and CDX2 (Extended Data Fig. 5e). Second, we asked whether depletion of USP7 or HU treatment can improve developmental efficiency after nuclear transfer (NT), as a readout for expanded cell potency as previously described for 2CLCs^7,21,31^. We performed NT into enucleated mouse oocytes using 2CLCs induced after siRNA for USP7 or upon HU treatment as donor. Remarkably, the number of embryos that cleaved to the 2-cell stage and formed hatching blastocysts was greatly increased when USP7-depleted or HU-treated green fluorescent protein-positive (GFP^+^) cells were used as donors, compared with controls (Fig. 3l,m, Extended Data Fig. 5f and Supplementary Table 3). These findings are in line with the known increased reprogrammability of control 2CLCs^7^. These experiments using USP7-depleted and HU-induced 2CLCs as donors suggest that they correspond to endogenous 2CLCs^6,7^ in terms of cellular potency. Thus, we conclude that reducing replication fork speed generates cells with a higher propensity to be reprogrammed upon NT.

### 2CLCs display distinctive changes in RT

**Fig. 4:**
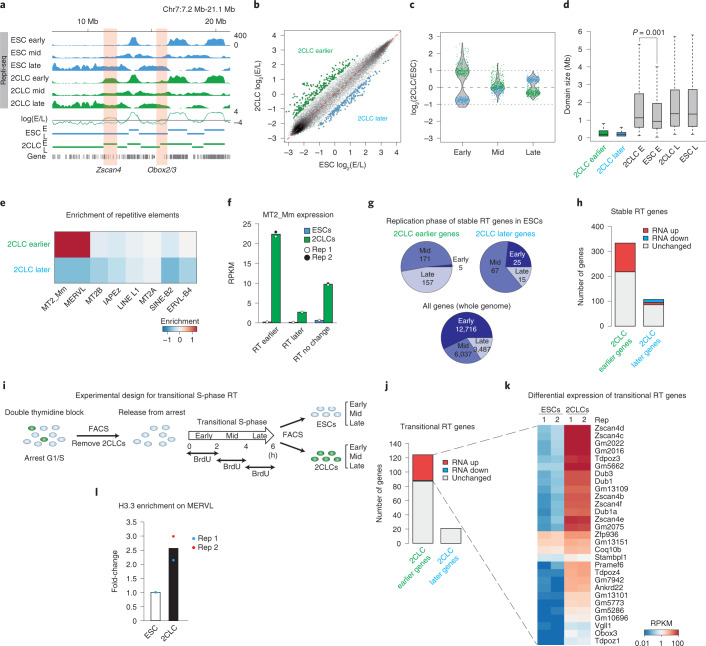
2CLCs display changes in RT and slowing replication fork speed promotes reprogramming to totipotency during SCNT. **a**, Repli-seq tracks indicating early:late ratio as log_2_(E/L) for ESCs and 2CLCs. Horizontal lines indicate early (E) and late (L) replicating domains. Orange highlights regions of differential RT. **b**, Comparison of log_2_(E/L) between 2CLCs and ESCs at 100-kb bins across the genome. Green and blue points are regions of differential RT (twofold cutoff). **c,** Violin plot of log(fold differences) between 2CLSs and ESCs at early, mid and late S-phase over twofold differential log_2_(E/L) 100-kb bins. **d**, Early replicating domains are larger in 2CLCs. Boxplot of domain sizes for differential RT shows early or late domains from two biological replicates. Boxes show IQR between first and third quartiles, horizontal line shows the median and whiskers show *Q*3 + 1.5 × IQR and *Q*1 − 1.5 × IQR. Statistical significance comparing domain sizes was by two-sided Student’s *t-*test. **e**, Genomic regions replicating earlier in 2CLCs are enriched in MT2_Mm and MERVL. Heatmap showing log_2_(fold enrichment) of repeats within earlier and later RT regions. **f**, Barplot of average expression (RPKM (reads per kilobase per million mapped reads)) in ESCs and 2CLCs of MT2_Mm repeats, the replication of which showed earlier, later or no shift in 2CLCs. Points indicate values for biological RNA-seq replicates. **g**, Shift of most genes to earlier replication in 2CLCs replicating in mid or late S-phase in ESCs. Proportions are shown by the numbers of genes according to their replication profile in ESCs, for the gene sets that shifted to earlier or later timing in 2CLCs. **h**, Genes replicating earlier in 2CLCs tend to be upregulated in 2CLCs. The barplot shows number of genes shifting to earlier or later RT in 2CLCs according to their expression changes (115 upregulated, 0 downregulated and 218 unchanged). **i**, Strategy to map replication timing in emerging 2CLCs during their transitional S-phase. **j**, Barplot depicting number of genes shifting to earlier or later RT during the transitional S-phase, during which 2CLCs emerge, according to their expression changes (29 upregulated, 0 downregulated and 95 unchanged) in 2CLCs. **k**, Most upregulated genes showing replication shift to earlier in transitional S-phase are repressed in ESCs. Heatmap depicts differential gene expression of genes shifting to earlier RT during the transitional S-phase. RPKM derive from two biological replicates. **l**, Relative H3.3 enrichment in ESCs and 2CLCs expressing SNAP-tagged-H3.3 analyzed by qPCR CUT&RUN. Dots represent biological replicates. Source data

**Extended Data Fig. 6:**
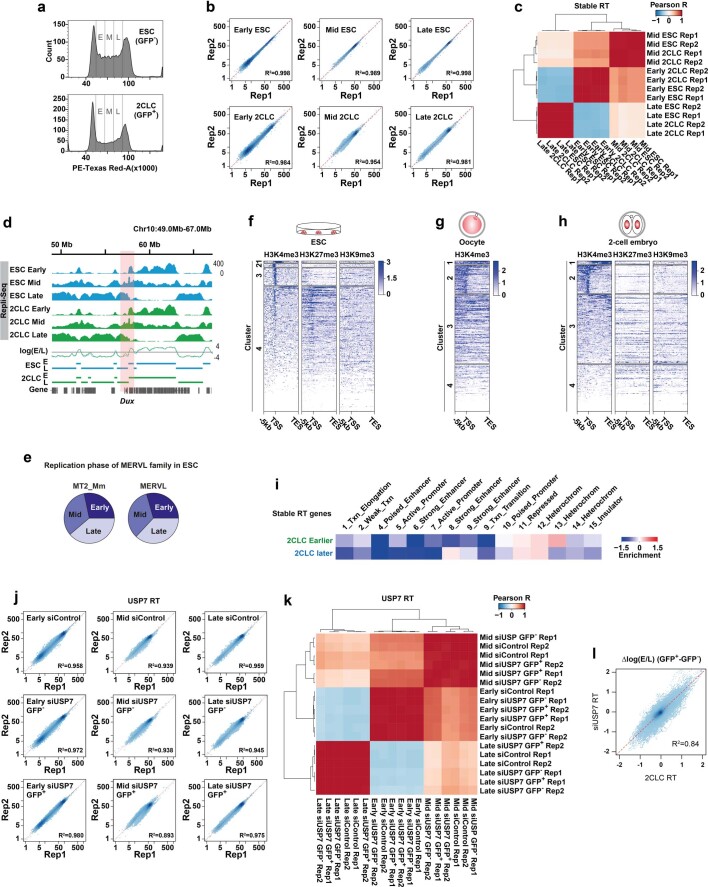
Genome-wide analysis of replication timing (RT) in 2CLCs. **a**. Collection of early, mid, and late S-phase of ESCs and 2CLCs. Each subpopulation of S-phase in ESCs (top) and 2CLCs (bottom) was sorted based on their DNA content upon propidium iodide staining. **b**. Scatter plots of read density at 100 Kbp bins across the whole genome between Repli-seq replicates of ESCs (GFP^−^ cells) and 2CLCs (GFP^+^ cells). Pearson R^2^ is indicated. **c**. Heatmap depicting Pearson R correlation based on read density at 100 Kbp bins across the genome for the indicated samples. **d**. Repli-seq tracks around the Dux locus at early, mid, and late S-phase in ESCs and 2CLCs indicating early to late ratio as log2(E/L). **e**. Distribution of MERVL elements (MT2_Mm and MERVL_int) according to early, mid, and late replication regions in ESCs. **f**. Heatmaps of histone modification densities in ESCs in the 5-Kbp vicinity of gene bodies for 333 genes with differential replication timing. **g**. Heatmaps of histone modification densities in oocyte in the 5-Kbp vicinity of gene bodies for 333 genes with differential replication timing. **h**. Heatmaps of histone modification densities in 2-cell embryos in the 5-Kbp vicinity of gene bodies for 333 genes with differential replication timing. **i**. Log2-fold enrichment of ENCODE chromatin states among genomic regions shifting to earlier (top) and later (bottom) replication in 2CLCs. **j**. Scatter plots of read density at 100 Kbp bins across the genome between Repli-seq replicates of control siRNA-transfected ESCs, and GFP^+^ (USP7KD-induced 2CLC) and GFP^−^ cells following USP7 depletion. Pearson R^2^ is indicated. **k**. Heatmap depicting Pearson R correlation based on read density at 100 Kbp bins across the genome in each S-phase of control siRNA transfected ESCs, and GFP^+^ (USP7KD-induced 2CLC) and GFP^−^ cells following USP7 depletion. **l**. Usp7KD-induced 2CLCs display changes in replication timing similar to 2CLCs, when compared to ESCs. Scatter plot shows a high degree of correlation (R^2^=0.84) between the differences in Early to Late ratio for 2CLCs vs ESCs (x-axis) and for Usp7KD-induced 2CLC vs GFP^−^ cells following USP7 depletion, among 100 Kbp bins across the genome.

Next, we explored the possible consequences of the differences in fork speed between ESCs and 2CLCs. We hypothesized that a slower fork speed, known to entail an increase in active origins to maintain the duration of the S-phase^32^, may result in changes in RT. Mammalian cells display an orderly program for replicating their genome in units of around 400–800 kb, which are coordinately replicated at determined times during the S-phase^33–35^. Early replication often correlates with the transcriptional potential of a gene^36^, although a causal relationship between RT and gene expression has not been firmly established. We first investigated whether 2CLC reprogramming entails a change in RT. We generated genome-wide RT maps from sorted ESCs and 2CLCs in early, mid and late S-phase (Extended Data Fig. 6a–c). A survey over the genome browser revealed specific gene regions shifting to earlier RT in 2CLCs. These included ‘2C’-specific genes such as *Zscan4*, *Obox2/3* and *Dux* (Fig. 4a and Extended Data Fig. 6d). Inquiry into the genomic regions shifting RT between the two cell types^37^ revealed changes across the genome in the replication timing of 2CLCs, compared with ESCs (Fig. 4b). These changes represented approximately 3% of the genome, and most occurred by shifting at early S-phase (Fig. 4c), in line with our observations above suggesting that the early S-phase is critical for 2CLC emergence. These changes corresponded primarily to enlarged early replication domains in 2CLCs, leading to larger replication domains in the early S-phase in 2CLCs, compared with ESCs (Fig. 4d). In addition, domains shifting to earlier RT were enriched for MERVL sequences, in particular the MERVL promoter (LTR, MT2_Mm) and internal sequences (MERVL), but not for other endogenous retroviruses, LINE-1 or SINE-B2 elements (Fig. 4e). This shift to earlier RT matches a higher expression of MERVL elements in 2CLCs compared with those that change RT or shifted to a later pattern of replication (Fig. 4f and Extended Data Fig. 6e). We next examined the genes that change RT in 2CLCs. We identified 440 genes that shifted in their RT profile, most of which changed to an earlier phase (76%; *n* = 333 genes) (Supplementary Table 4). Among them, most changed from mid- and late RT in ESCs to earlier replication in 2CLCs (98%; *n* = 328 genes) (Fig. 4g). These genes included genes from the ‘2C’ program, such as *Zscan4* and *Dux* (Fig. 4a and Extended Data Fig. 6d). Approximately a quarter of the RT-changing genes shifted to a later pattern of replication (*n* = 107 genes). Among the genes that changed RT, only 30% (*n* = 136) were differentially expressed in 2CLCs compared with ESCs and most of these shifted to an earlier RT (Fig. 4h). This suggests that only a fraction of the changes in RT of 2CLCs is concordant with changes in gene expression. To address the chromatin status of the genes that shift RT, we analyzed ESC and 2-cell-stage embryo chromatin immunoprecipitation followed by sequencing (ChIP-seq) datasets. In general, RT genes displayed enrichment of H3K4me3 at promoters or had bivalent signatures (Extended Data Fig. 6f–h), in agreement with their expression state in ESCs and 2-cell-stage embryos^38^. Some were enriched with H3K9me3 (Extended Data Fig. 6f–h) and the ENCODE term ‘heterochromatin’ was significantly over-represented in the RT regions that shift to earlier RT in 2CLCs (Extended Data Fig. 6i). This is in line with our observation that MERVL shifts to earlier RT in 2CLCs. RT profiles in 2CLCs induced by *Usp7* knockdown (Extended Data Fig. 6j,k and Supplementary Table 5) displayed overall a similar RT profile compared with endogenous 2CLCs (Extended Data Fig. 6l), suggesting that the changes in replication fork speed during the S-phase, elicited by USP7 depletion, lead to a similar change in the RT profile in 2CLCs. Thus, we conclude that 2CLCs display a distinctive RT profile, characterized by changes to early replication of MERVLs and part of the 2C program. Importantly, as an excess number of origins are licensed in G1 than are used during the S-phase^39,40^, these data are consistent with our observations indicating that entry into early S-phase is important, and suggest that additional origins may fire during early S-phase to promote 2CLC emergence.

### MERVL shift to earlier replication during 2CLC reprogramming

**Extended Data Fig. 7:**
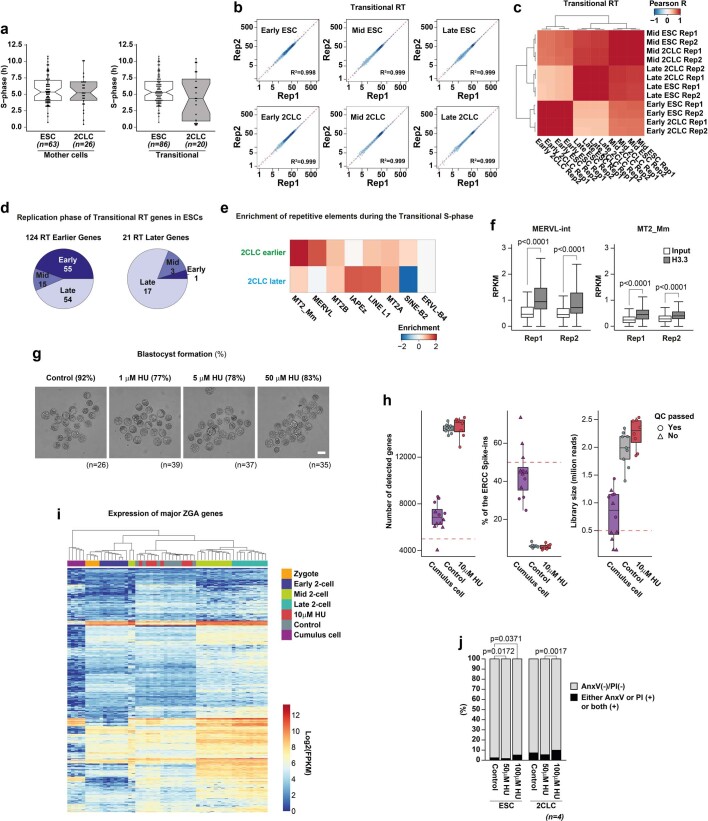
Genome-wide analysis of replication timing (RT) in the transitional S-phase during which 2CLC emerge. **a**. S-phase length in mother cells of 2CLCs and ESCs and in ESCs and 2CLCs during the transitional S-phase. Boxplots: median (middle line) IQR (boxes) and extent of data without outliers (whiskers,>1.5x IQR). Notches extend to + /−1.58xIQR/sqrt(n), indicating confidence intervals. Dots are individual measurements arranged in 0.2 h bins. **b**. Scatter plots of read density at 100 Kbp bins across the genome between Repli-seq replicates of the transitional S-phase of cells transitioning from ESC to 2CLC. Pearson R^2^ is indicated. **c**. Pearson R correlation heatmap based on read density at 100 Kbp bins across the genome in each S-phase of cells transitioning from ESC to 2CLC. **d**. Pie charts of numbers of genes that replicate in early, mid and late S-phase in ESCs, for gene-sets whose replication shifted to earlier and later timing during the transitional S-phase in emerging 2CLCs. **e**. Enrichment of repeat elements across genomic regions changing to an earlier and later replication timing during the transitional S-phase at which 2CLCs emerge. **f**. H3.3 enrichment at MERVL-int and MT2_Mm repeats in 2-cell embryos. Reads were normalized by sequencing depth and length, data from two biological replicates shown separately as 25th and 75th percentiles (box), median (line) and smallest and largest values within 1.5×IQR of the hinge (whiskers). Statistical analyses against the input were with two-sided Wilcoxon-signed-rank test. **g**. Developmental progression of fertilized embryos upon HU treatment. Zygotes collected at 17-18 h post-hCG were treated with HU until 48 h posthCG. Embryos reaching the blastocyst stage (%) are indicated; n: number of embryos analyzed. Scale bar, 100 μm. **h**. RNAseq quality control (QC) metrics for nuclear transferred embryos (control and 10μM HU-treated) and single cumulus cells. QC thresholds (red dotted lines) are indicated; samples failing QC (triangles) were discarded. Boxplots show median and IQR; whiskers depict the smallest and largest values within 1.5×IQR. **i**. Heatmap with expression of ZGA genes upon nuclear transfer compared to *in vivo* derived embryos. **j**. Cell death analysis by dual Annexin-V and propidium iodide (PI) staining following HU treatment. Cells positive for either or both Annexin-V and PI were considered dead. Statistical analyses: two-sided Student’s *t*-test.

Next, to address whether changes in RT temporally precede changes in cell fate, we devised an approach to map RT of emerging 2CLCs in the S-phase during which they transition toward 2CLCs, which we referred to as the ‘transitional’ S-phase (Fig. 4i). Our experimental design enabled us to analyze all cells that would undergo reprogramming in a synchronized fashion during the S-phase. Notably, the length of the S-phase of the transitioning cells, albeit variable, did not differ significantly in either the ‘mothers’ of the 2CLCs or the emerging 2CLCs themselves, compared with ESCs (Extended Data Fig. 7a), which enabled direct comparison of the RT profiles in both cell types. Our transitional RT datasets showed good correlation among replicates (Extended Data Fig. 7b) and revealed minor changes in RT compared with ESCs (Extended Data Fig. 7c), similar to the RT datasets of ‘stable’ 2CLCs. Analysis of the genes, which shift RT during the transitional S-phase, revealed 6 genes that shifted earlier with at least a 2-fold difference between ESCs and 2CLCs, and 145 genes with at least a 1.5-fold difference (Methods; Extended Data Fig. 7d and Supplementary Table 6). The fact that RT analysis in stable 2CLCs displays a higher number of genes that change in RT compared with the transitional RT dataset could indicate that part of the RT program of 2CLCs changes during the transitional S-phase, during which 2CLCs emerge, but another portion is achieved and consolidated once 2CLCs have been reprogrammed. Notably, most genes that shift to earlier RT during this transitional S-phase are not expressed in ESCs and become highly upregulated in 2CLCs (Fig. 4j,k)^7^. These genes belong to both the Zscan4-signature and the 2C signature^6,10,41,42^. Likewise, MERVL elements were enriched in domains shifting to earlier RT before 2CLC emergence (Extended Data Fig. 7e). As we detected differences in RT already during the transitional S-phase before 2CLC emergence, these data suggest that changes in RT of a subset of 2C genes and MERVL elements occur before changes in cell fate and transcriptional profile.

To address how a change in RT could potentially affect MERVL expression, we investigated their chromatin status because alteration of RT can disrupt chromatin modifications^43^. To restore the chromatin template after replication and preserve the corresponding epigenetic information, the replication machinery interacts with and recruits chromatin modifiers and remodelers^44^. Distinct chromatin proteins associate with the replication machinery in early versus late S-phase^45,46^. For example, ‘new’ histone H3.3 is known to be enriched at nascent chromatin specifically in the early S-phase^47^. H3.3 is associated with transcriptionally active chromatin and is incorporated throughout the cell cycle^48,49^. Thus, we investigated the distribution of H3.3 at MERVL. CUT&RUN for H3.3 indeed revealed that H3.3 is enriched at MERVL in 2CLCs, compared with ESCs (Fig. 4l). H3.3 is also enriched at MERVL in 2-cell-stage embryos (Extended Data Fig. 7f), coincident with the onset of MERVL expression^50^. Thus, a change in RT is associated with H3.3 enrichment at MERVL upon 2CLC emergence.

### Slowing replication promotes reprogramming during SCNT

**Fig. 5:**
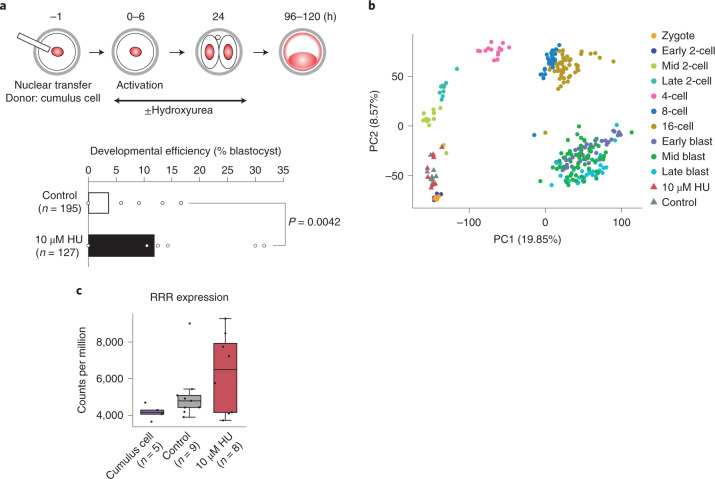
Improvement of the developmental potential of SCNT-derived embryos. **a**, SCNT embryos from cumulus cells treated with HU for 24 h after NT. Reprogramming efficiency was estimated by calculating the developmental rate of NT-derived embryos to the blastocyst stage. Barplots indicate the percentage of developmental efficiency of ten (control) and seven (10 μM HU) independent experiments. Each dot indicates the percentage obtained in each of these experiments and *n* indicates the total number of activated oocytes analyzed. Statistical analyses were performed using the *z*-score test for two population proportions (two tailed). **b**, Principal component (PC) analysis depicting the transcriptional profile of all NT embryos 28 h after activation, analyzed by single embryo RNA-seq, in comparison with wild-type embryos^38^. Note that NT-derived embryos cluster at the corresponding developmental time at which they were collected, indicating transcriptional reprogramming. **c**, Boxplot depicting the expression levels across RRRs in cumulus cells and control and HU-treated NT embryos. Each dot represents individual embryos (biological replicates). The boxplots indicate the first and third quartiles as the lower and upper hinges and the whiskers extend to the lowest and highest value no further than 1.5 × IQR.

Finally, we sought to address the functional relevance of the replication dynamics and fork remodeling for reprogramming to totipotency. Terminally differentiated somatic cells can be reprogrammed to totipotency upon transplantation into enucleated oocytes^51,52^. However, this process is inefficient and often development beyond the 2-cell stage is considered to be a bottleneck^31^. Considering the slower fork speed that we observed in 2-cell-stage embryos, we addressed whether reducing fork speed improves somatic cell nuclear transfer (SCNT) efficiency using cumulus cells as donors. In normal fertilized embryos, HU treatment did not affect developmental progression (Extended Data Fig. 7g). Remarkably, HU treatment greatly increased SCNT efficiency, leading to significantly higher developmental rates compared with the controls (3.5-fold, *P* = 0042; Fig. 5a). RNA-seq analysis of NT embryos indicated that cloned embryos have effectively reset their transcriptional landscape, including activation of zygotic genome activation genes and importantly, also, of ‘reprogramming resistant regions’ (RRRs)^31^ (Fig. 5b,c and Extended Data Fig. 7h,i). Thus, these results suggest that manipulating replication fork speed can improve cloning and facilitate reprogramming to totipotency.

## Discussion

The overall rate of DNA synthesis is controlled by altering the rate at which individual replication forks synthesize DNA and/or changing the total number of active forks in the S-phase. In other vertebrates, such as *Xenopus*, embryonic cells divide extremely fast when the embryo goes from 50 to >5,000 cells, with S-phase lasting ~14 min at the earliest measured stage^53^. Although fork speed has not been determined before the midblastula transition, work with egg extracts supports a model whereby a high density of randomly positioned origins ensures genome duplication within this very short time^54,55^. DNA combing at the ribosomal DNA locus also revealed that frequency of initiation decreases from the early blastula onward^56–58^. However, similar analyses have not been done in mammals. Our data in the mouse indicate that the mammalian embryo replicates its DNA with low speed in the first three cell cycles after fertilization.

Our data suggest a working model whereby slower fork speed and the concomitant higher ratio of origins to forks enable a shift of RT of specific genomic regions, which are enriched in MERVL, toward early S-phase. Early replication may enable the recruitment of factors preferentially associated with replicative chromatin in early S-phase compared with late S-phase^46,47^. We propose that a change in RT provides a window of opportunity to alter the chromatin template toward transcriptionally permissive chromatin, for example, through the incorporation of the histone variant H3.3 (ref. ^47^). Indeed, H3.3 can be deposited during the S-phase^59,60^ and therefore changes in the distribution of H3.3 can potentially occur as a consequence of earlier replication. This is consistent with our data showing that MERVLs, which shift toward earlier RT, become highly expressed in 2CLCs and with data indicating that H3.3 enrichment at MERVL in the 2-cell-stage embryos is dependent on DNA replication^50^. This, in turn, may facilitate the expression of 2C genes driven by MERVL^6,9,21,61^. Indeed, H3.3 is required for de novo global transcription and embryonic development^62^. Molecular studies to determine the position and the number of origins used are currently impossible in embryos or 2CLCs, primarily because techniques to identify origins require amounts in the millions of cells. Identifying the mechanisms for origin firing during reprogramming and early development will demand further study and the development of low-input protocols. Our work contributes to the molecular characterization of 2CLCs, for which similarities to and differences from the 2-cell-stage embryo have started to emerge^61,63–65^.

DNA damage induces *Zscan4* expression^66^ and has recently been shown to promote expression of *Dux* through direct transactivation by p53 (ref. ^67^). It is interesting that, upon DNA damage, the DNA-damage response (DDR) kinases ATR (ataxia telangiectasia and Rad3 related) and ATM (ataxia telangiectasia mutated) are required for DNA-damage-induced 2CLCs^67^. Earlier work documented that aphidicolin treatment, leading to increased phosphorylation of CHK1 in ESCs, induces *Zscan4* and *MERVL* expression^68^. However, although chemical inhibition of ATR partly reduced the extent of ZSCAN4 activation, this was not the case in ATR-deficient ESCs^68^. Although checkpoint activation and DNA damage can induce 2CLCs^67^, 2CLC emergence can also occur without checkpoint activation^69^. It is noteworthy that most studies on the role of checkpoint activation in 2CLC induction are based on experimental induction of DNA damage, but only few have been performed in unperturbed conditions. Our work in naturally cycling 2CLCs, demonstrating the lack of detectable increase in γH2A.X in 2CLCs and that depletion of several checkpoint mediators does not impact the number of 2CLCs^10^, suggests that DDR is not necessarily always involved in this process. This is in line with recent findings by Grow et al., which support both p53-dependent and p53-independent mechanisms for regulating DUX^67^.

Overall, we suggest that regulation of fork speed can act as a fate determinant factor. Thus, our work highlights fundamental features of DNA replication in reprogramming cell fate.

## Methods

### Embryo collection and culture

All mouse experiments were approved by the Ethics Committee of the Université de Strasbourg (Com’eth Institute of Genetics, Molecular and Cellular Biology) and performed under the compliance of either French legislation or the government of Upper Bavaria. F1 female mice (C57Bl/6J × CBA) aged <10 weeks were superovulated by intraperitoneal injection of 10 U of human chorionic gonadotropin (hCG) followed by 10 U of pregnant mare serum gonadotropin 48 h later, and then mated with F1 male (C57Bl/6J × CBA) mice. Zygotes were collected from the oviduct, placed in drops of KSOM (potassium-supplemented SOM) and cultured at 37 °C with 5% CO_2_ as previously described^70^.

### ESC culture

Mouse E14 ESC lines were cultured in Dulbecco’s modified Eagle’s medium (DMEM) with GlutaMAX (Invitrogen) containing 15% fetal calf serum, 2× leukemia inhibitory factor, penicillin–streptomycin, 0.1 mM 2-mercaptoethanol, 3 μM CHIR99021 (GSK3β inhibitor) and 1 μM PD0325901 (MEK inhibitor) on gelatin-coated plates unless otherwise stated.

### FACS

For isolation and quantification of 2CLCs, cells were washed twice with phosphate-buffered saline (PBS) and treated with 0.25% trypsin. After neutralization with ESC medium, cells were collected by centrifugation and the dissociated single cells were resuspended in ESC medium. To calculate the population of 2CLCs, we counted turbo GFP^+^ ESCs after exclusion of dead and doublet cells based on the forward and side-scatter profiles. After sorting, cells were collected in normal culture medium and kept at 4 °C. For collection of cells in G1-phase in Fig. 2e and Extended Data Fig. 3e, we sorted the mCherry-hCdt1(1/100)Cy(−)-positive, iRFP-hGeminin(1/110)-negative subpopulation based on their fluorescence. For cell cycle analysis, the dissociated single cells were fixed with 70% ethanol for 30 min. After treatment with 250 µg ml^−1^ of RNase A (Thermo Fisher Scientific) for 5 min, cells were treated with 50 μg ml^−1^ of propidium iodide (PI) to stain DNA. For the cell death analysis in Extended Data Fig. 7j, harvested cells were incubated with Annexin-V, APC conjugate (A35110) for 15 min at room temperature in binding buffer (10 mM Hepes, pH 7.4, 140 mM NaCl, 2.5 mM CaCl_2_), according to the manufacturer’s protocol. Cells were subsequently washed with binding buffer and stained with 0.5 μg ml^−1^ of PI for 15 min on ice. Sorting was performed on a BD Biosciences FACSAria III and FACSMelody. Percentage of 2CLCs was calculated using FACSDiva and FACSChorus and the analysis of other FACS data was performed using FlowJo software.

### DNA fibers in embryos and ESCs

DNA fibers were prepared as described^12,71^, which we applied to low cell numbers. Embryos and ESCs transfected with siRNA or treated with HU were sequentially pulse labeled with 25 μM 5-iodo-2′-deoxyuridine (IdU; Sigma-Aldrich) and 50 μM 5-chloro-2′-deoxyuridine (CldU; Sigma-Aldrich) for 30 min each and collected. Labeled cells were lysed and DNA fibers were stretched on to the slide glass by tilting. The fibers were fixed in methanol:acetic acid (3:1), then denatured with 2.5 M HCl for 1 h, neutralized with PBS and blocked with 1% bovine serum albumin/0.1% Tween-20 in PBS. CldU and IdU tracks were detected with anti-bromodeoxyuridine (anti-BrdU) antibodies (described in Supplementary Table 7) recognizing CldU and IdU, respectively, and appropriate secondary antibodies. After the detection of IdU and CldU tracks, DNA was detected using an antibody against single-stranded DNA and the corresponding secondary antibody. 2-cell embryos in early S-phase, mid S-phase and late S-phase, and 4-cell embryos, 8-cell embryos and blastocysts were collected at 35, 37, 39, 53, 70 and 96 h post-hCG injection, respectively. Images were acquired on a Leica SP8 confocal microscope using a ×40 Plan/Apo NA1.3 oil immersion objective (Leica) at 2,048 × 2,048 pixels^2^ at an effective pixel size of 142 nm. To calculate fork speed, we used the established conversion 1 μm = 2 kb (ref. ^72^). Analysis of DNA fibers was performed by two different researchers using a customized image analysis pipeline that consisted of three steps: (1) localization of fibers in confocal images, (2) detection of branch modes in each fiber and (3) statistical analysis of different fiber parameters (for example, pattern proportion, branch length). As a prerequisite step, we employed masks to select regions of interest in the images, which contained a sufficient number of fibers to be analyzed. Briefly, for the fiber localization, we used a vessel detection algorithm, using a space-scale local variational approach, followed by a morphological reconstruction to extract the median line by B-spline fitting. To overcome issues of noise and signal heterogeneity, we implemented a structure reconstruction with a spatially variant morphological closing^73^. The process uses a small segment (at least the size of disconnection, for example, 20 pixels) as a structuring element. The map is then thresholded to a certain value (typically 0.5) and single fibers are identified separately by a connected component algorithm. Then, the skeletons of the fibers were identified by a morphological thinning and fitted to achieve subpixel accuracy. To detect patterns in the extracted fibers, we used a branch detection strategy. Briefly, intensity profiles from both channels were sampled along the median line. As the channels were not directly comparable in absolute intensity value, the logarithm of their point-wise intensity ratio was used instead. We used regression tree structures in combination with the CART algorithm^74^, which uses a partitioning algorithm to detect the patterns of the DNA fibers. Subsequently, a semi-automated step to verify fiber detection and features was implemented manually. The fiber analysis software is written in Python and is available at https://github.com/IES-HelmholtzZentrumMunchen/dna-fibers-analysis. To calculate the IOD, we manually selected sufficiently long fiber stretches from our DNA fiber dataset in the DNA channel, which encompassed several IdU/CldU boundaries. To facilitate the analysis, we generated a Fiji (ImageJ) macro to open the regions of interest in the images and applied the ImageJ ‘Straighten’ function with a width of 19 pixels to convert bent fibers into approximately two-dimensional images, where the channel intensities were interpolated along the *x* axis. In the stretched fiber images, we then manually selected all identifiable IdU/CldU boundaries. The remaining analysis was performed in R. We first calculated from the *x* coordinates of the boundaries all origin positions by averaging between two adjacent boundary points. We then determined the pairwise difference between origins to obtain the IOD. IOD and boxplots were created using the ggplot2 library in R.

### Cell cycle synchronization and drug treatment

For all G1/S synchronization with thymidine, a double thymidine block was used as follows: cells were incubated for 12 h with 2.5 mM thymidine, released for 9 h after washing out the thymidine, and then blocked again with 2.5 mM thymidine for 14 h to arrest all cells at the beginning of S-phase. For release experiments (Figs. 2a–c, 3g,h and 4i–k and Extended Data Fig. 2a–c,f), cell cycle arrest was subsequently released with two washes of thymidine-free medium. After release, cells were harvested at 1-h intervals or treated with 1 μM aphidicolin or 2.5 mM thymidine for 6 h. For other drug treatments (Fig. 2d and Extended Data Figs. 2g,h, 3a–d and 4i–k), the following inhibitors and concentrations were used: CDC7 inhibitor (PHA-767491; 10 μM), CDK1 inhibitor (RO-3306; 10 μM), PLK1 inhibitor (BI-6727; 500 nM) were used to synchronize cells for 8, 10 and 4 h, respectively. In Fig. 2e and Extended Data Fig. 3e, cells in G1-phase were sorted by FACS based on their FUCCI reporter system as described in FACS. After sorting, cells were plated under normal culture conditions or with medium supplemented with 10 μM CDC7 inhibitor. After culturing for 6 h, cells were analyzed by FACS to calculate the number of 2CLCs.

### RNA-seq

Forty-eight hours after transfection of siRNA for control and USP7, cells were FACS sorted into ESCs and 2CLCs based on the GFP fluorescence, reflecting the 2C::tbGFP reporter activity. Total RNA was extracted using PicoPure RNA Isolation Kit (Thermo Fisher Scientific) and treated with turbo DNase (Life Technologies). Two biological replicates were prepared for each sample and their quality was checked using the 2100 Bioanalyzer with the RNA 6000 Nano Kit (Agilent). Libraries for strand-specific sequencing were created with a TruSeq Stranded Total RNA Library Prep Human/Mouse/Rat (Illumina) and IDT for Illumina-TruSeq RNA UD Indexes (Illumina) according to the manufacturer’s protocol. Excess primers were removed through a purification step using AMPure XP beads (Agencourt Biosciences Corporation). The quality and quantity of the complementary DNA libraries were verified with the 2100 Bioanalyzer using the High Sensitivity DNA Kit (Agilent). Sequencing was carried out on an Illumina HiSeq 4000 (Illumina) with a 150-bp paired-end protocol according to Illumina’s instructions.

### NT with 2CLCs and ESCs

NT was performed as described^51^ with slight modifications^75,76^. Metaphase II-arrested oocytes were collected from superovulated F1 female mice (C57Bl/6J × CBA) aged <10 weeks and cumulus cells were removed using hyaluronidase. Oocytes were enucleated in a droplet of M2 medium containing 5 μg ml^−1^ of cytochalasin B (CB) using a blunt Piezo-driven pipette. After enucleation, the spindle-free oocytes were washed extensively and maintained in CZB medium up to 2 h before nucleus injection. Nuclei of ESCs and 2CLCs (E14 background, originally derived from 129/Ola mouse strain) cultured in serum/leukemia inhibitory factor (nontreated, siControl-transfected, siUSP7-transfected or HU-treated cells) were collected by FACS based on their GFP fluorescence and size, and were aspirated in and out of the injection pipette to remove the cytoplasmic material and then injected into enucleated oocytes. The reconstructed oocytes were cultured in CZB medium for 1 h and activated for 6 h in Ca^2+^-free CZB medium containing 10 mM Sr^2+^ and 5 μg ml^−1^ of CB. After activation, the reconstructed embryos were cultured in KSOM at 37 °C under 5% CO_2:_air for 5 d and subsequently checked for their developmental efficiency. Note that, although most NT protocols employ Trichostatin A, we purposely refrained from using Trichostatin A to avoid confounding effects due to potential alterations to chromatin structure.

### SCNT

SCNT was performed using cumulus cells as donors. For these experiments, we used two different F1 mouse strains to provide robustness and validation: C57BL/6J × DBA/2J and C57Bl/6J × CBA. The same protocol as for 2CLCs and ESCs was used, with slight modifications. Briefly, MII oocytes were collected and enucleated in CZB medium and then allowed to recover in KSOM until they were used for NT. The nuclei of donor cumulus cells were injected into the enucleated oocytes using a Piezo-driven micromanipulator. After reconstruction, oocytes were cultured for 1 h in KSOM and activated for 6 h in KSOM containing 10 mM Sr^2+^ and 5 μg ml^−1^ of CB supplemented with 2 mM (ethylenebis(oxonitrilo))tetra-acetate^77^. Embryos were then randomly distributed in medium with or without HU (10 μM), which was replaced by fresh medium without HU after 24 h. Experimental design and scoring were double blinded. The SCNT data derived from the two mouse strains were verified for consistency and the sum of the compiled data is shown in Fig. 5a.

### Replication timing

For the stable RT and USP7 RT, synchronously cycling cells were pulse labeled with the nucleotide analog BrdU for 2 h, respectively. Cells were sorted into early, mid and late S-phase fractions, 20,000 cells each, on the basis of DNA content using FACS. For the transitional RT, existing 2CLCs were removed after double thymidine block. After release from G1/S arrest, ESCs were treated with BrdU for 2 h during the specific time windows indicated in Extended Data Figure 4i (0–2 h for early S-phase, 2–4 h for mid S-phase or 4–6 h for late S-phase). ESCs and newly emerged 2CLCs were sorted by FACS based on the 2C::tbGFP fluorescence 6 h after release from G1/S block, and genomic (g)DNA was isolated from each condition (that is, early, mid or late S-phase for ESCs and 2CLCs) using sodium dodecylsulfate–proteinase K buffer and purified by phenol–chloroform extraction. The gDNA was fragmented using the Covaris sonicator to obtain fragments of 700 bp on average. The sheared, BrdU-labeled DNA from each fraction was immunoprecipitated using 0.5 μg of mouse anti-BrdU antibody followed by addition of 50 μl of precleared Dynabeads coupled to sheep anti-mouse immunoglobulin G (Invitrogen). The immunoprecipitated pellet was digested overnight with proteinase K and purified by phenol–chloroform extraction. RT libraries were prepared based on Accel-NGS methyl seq library kit (Swift Biosciences) according to the manufacturer’s instructions. The BrdU-immunoprecipitated DNA was denatured and subjected to Adaptase reaction. This step was followed by an extension reaction with two cleanup steps utilizing Agencourt Ampure XP beads (Beckman Coulter). The eluate was subjected to a ligation step, followed by Ampure bead-mediated purification. Indexing PCR was performed at 98 °C for 30 s, 9 cycles at 98 °C for 10 s, 60 °C for 30 s and 68 °C for 60 s, followed by a 4 °C hold cycle. The PCR product was further purified by Ampure beads and eluted in a 20-μl volume using Tris–EDTA buffer provided by the manufacturer. The libraries were verified using Agilent 2200 Tape Station (Agilent) utilizing DNA high-sensitivity tape (Agilent). Up to 12 libraries were pooled together after Qubit quantification with Qubit DNA HS assay kit (Thermo Fisher Scientific) and loaded into Nextseq 500/550 high-output cartridge (Illumina) for 75 cycles of single-end sequencing.

### RT analysis

Repli-seq reads from early, mid and late time points of S-phase were mapped to the reference mm9 genome using BWA^78^ and counted over 100-kb genomic bins across the genome, followed by the Loess smoothing of bin counts as previously described^37^. The E/L was calculated from the read counts in early and late S-phase. Regions with differential RT between ESCs (GFP^−^) and 2CLCs (GFP^+^) cells were determined based on 2-fold (or 1.5-fold for the transitional S-phase) cutoff of change in E/L ratio. Domains of early and late replication were identified using the DNAcopy package^79^. Genes were classified as early, mid or late replicating based on the stage of S-phase with the highest read density over the gene body. This three-stage classification was highly consistent with the traditional E/L based only on the reads from early and late stages.

### Single embryo RNA-seq and library preparation

Control and HU-treated (10 μM) nuclear transferred embryos were cultured until 28 h after activation, at which point a representative proportion of embryos was collected, washed with PBS, placed in tubes with 1× Clontech lysis buffer (Z5013N) containing ERCC RNA Spike-In Mix (Invitrogen) and flash-frozen in liquid nitrogen. RNA-seq was carried out using the SMART-seq2 protocol^80^ and subjected to paired-end sequencing on a Nextseq 500 (Illumina) platform. A total of nine control and eight HU-treated embryos derived from two independent experiments were sequenced. In parallel, we collected 12 single cumulus cells used as donors and processed them for RNA-seq under identical conditions.

### Statistical analyses

To assess whether the data were normally distributed, we performed a Shapiro–Wilk test or *F*-test. For normally distributed data, we applied the Student’s *t*-test to perform pairwise comparisons between groups, as indicated throughout the figure legends; otherwise we applied the nonparametric Mann–Whitney (Wilcoxon’s rank-sum) test. The proportions of patterns from the DNA fiber data were analyzed by a binomial test in R (two-sample test for equality of proportions with continuity correction). Where data are shown as box-and-whisker plots, we followed the convention for boxplots^81^ (thick bar, median; boxes, IQR; whiskers, range without outliers; dots outside whiskers, outliers beyond 3× or 2× IQR). For datasets with unequal variance (Fig. 3l,m), we applied Welch’s test for unequal or unknown variances.

### Antibodies

Antibodies used in this work are described in Supplementary Table 7.

### Reporting Summary

Further information on research design is available in the Nature Research Reporting Summary linked to this article.

## Online content

Any methods, additional references, Nature Research reporting summaries, source data, extended data, supplementary information, acknowledgements, peer review information; details of author contributions and competing interests; and statements of data and code availability are available at 10.1038/s41588-022-01023-0.

## Supplementary information

Supplementary Information Supplementary Methods and references.

Reporting Summary.

Supplementary Tables Supplementary Table 1. List of differentially expressed genes on siUsp7 RNAi. EdgeR was used to call differentially expressed genes, which estimates statistical significance based on negative binomial statistics (two sided) followed by multiple testing adjustment using the Benjamini–Hochberg FDR. Supplementary Table 2. List of differentially expressed repeats on siUsp7 RNAi. EdgeR was used to call differentially expressed genes, which estimates statistical significance based on negative binomial statistics (two sided) followed by multiple testing adjustment using the Benjamini–Hochberg FDR. Supplementary Table 3. Developmental score of NT embryos. Supplementary Table 4. Genes showing RT changes in 2CLCs compared with ESCs. Supplementary Table 5. Genes showing RT changes on siRNA for *Usp7*. Supplementary Table 6. Genes showing changes in RT during the transitional S-phase. Supplementary Table 7. List of antibodies used in this work.

## Data Availability

The Repli-seq and RNA-seq data from the present study are available from the Gene Expression Omnibus database, accession nos. GSE136228 and GSE166338. Previously published RNA-seq datasets reanalyzed in the present study are available under accession nos. GSM1933935, GSM1625860, GSM1933937, GSM1625862, GSM1625864, GSM1625867, GSM1625868, GSM838739, GSM838738, GSM1625873, E-MTAB-2684 and GSM1933935. ChIP-seq datasets reanalyzed in the present study are available under accession nos. GSE73952, GSE97778, GSE73952, GSE23943 and GSE139527. Source data are provided with this paper. All other data supporting the findings of the present study are available from the corresponding author upon reasonable request.

## Source data

Source Data Fig. 3 Unprocessed western blots.
Source Data Fig. 4 Unprocessed western blots.

## Extended data

are available for this paper at 10.1038/s41588-022-01023-0.

## Code availability

All next-generation sequencing data were analyzed using standard programs and packages, as detailed in Methods. Code for DNA fiber analysis is available at: https://github.com/IES-HelmholtzZentrumMunchen/dna-fibres-analysis.
