# Supplementary material for: DNA replication fork speed underlies cell fate changes and promotes reprogramming
Source: Nat Genet. 2022 Mar 7;54(3):318–27. doi: 10.1038/s41588-022-01023-0 (PMC8920892; doi:10.1038/s41588-022-01023-0)
Supplement: Supplementary file 1 — Supplementary Methods and references. [file 41588_2022_1023_MOESM1_ESM.pdf]

nature portfolio

https://doi.org/10.1038/s41588-022-01023-0

Supplementary information

DNA replication fork speed underlies cell fate changes and promotes reprogramming

In the format provided by the authors and unedited

Supplementary Methods

Plasmid construction

The 2C::3xturboGFP reporter plasmid was described and characterised previously[1] and the the 2C::tdTomato reporter was acquired from Addgene (#40281) [2]. Both reporters are known to mark 2CLCs [1,2]. Expression plasmids that contain mCherry-hCdt1(1/100)Cy(-) or iRFP-hGeminin(1/110) were generated after amplification from mCherry-hCdt1(1/100)/pCSII-EF (RIKEN BRC, RDB15442) [3], iRFP-C1 (Addgene plasmid #54786), and ES-FUCCI (Addgene plasmid #62451) [4] and inserted into pCAG-Hyg or pCAG-bsd plasmid using the Ligation Mix (TaKaRa) or Gibson Assembly Master Mix (NEB). The cDNA encoding TIR1 was obtained from Addgene (#47328) and the amplified PCR fragment was ligated into the pCAG-Hyg. The Dux cDNA [5] was subcloned into pRN3P for *in vitro* transcription. The H2B-tdiRFP (Addgene #47884) has been described elsewhere [1]. H3.3 cDNA was subcloned into pSNAPf (NEB) and the resulting sequence encoding SNAP-tagged H3.3 was digested and inserted into pCAG-puro plasmid using Ligation Mix (TaKaRa).To construct the USP7 knock-in vector, the Auxin-Inducible Degron (AID), puromycin resistance, and polyA polyadenylation signal were PCR amplified. The P2A sequence was cloned from oligonucleotides. To construct the homology arms (HA) of the donor vectors, mouse genomic DNA was amplified using the following primers, and cloned into pBluescript II SK(+) : HAL: 5'-TTTGGCGGGCAGAAGATAATTG-3' and 5'-GTTGTGGATTTTAATTGCCTTTTC-3'
HAR: 5'-GGCGAGGGACAGTGCGTGGG-3' and 5'-AGAGGAGCCCCAGGAAGGGC-3'
Cas9 and sgRNA were expressed from the bicistronic pX330-U6-Chimeric_BB-CBh-hSpCas9 (Addgene plasmid #42230) vector. Targeting sequences of sgRNAs were designed using CRISPR direct (https://crispr.dbcls.jp/). Sense and antisense oligonucleotides of each sgRNA targeting sequence (USP7#1: TGACTTCCTTCCCGTGTCCA and USP7#2: TCCTTCCCGTGTCCAAGGCG were cloned into pX330 at the BbsI site. The comparison of USP7 expression levels between the parental clone and the USP7-AID clone by Western blot is shown in Extended Data Fig. 4n and indicates that the USP7-AID transgene leads to slightly lower expression of USP7 compared to the parental clone, and a consequent higher proportion of 2CLCs in the steady state population of this cell line.

Transfection and generation of stable cell lines

4μg of linearised plasmids, 40 pmol siRNA, and 2μg mRNA were transfected into 1-2x10^5 ESCs using 8μL Lipofectamine 2000 (Invitrogen), 8μL Lipofectamine RNAi MAX (Invitrogen), and 8μL Lipofectamine MessengerMAX (Invitrogen) according to the manufacturer's instructions with the following modifications, respectively: incubated DNA-, siRNA-, or mRNA-reagent mixture in Opti-MEM was mixed with cells suspended in culture medium (v/v = 1:1

ratio). Transfected cells were incubated for 5 minutes and were plated at a density of 5 to 12.5 × 10^3 cells/cm^2. siRNA of USP7, control, RRM1, and DUX were acquired from Dharmacon (D-52244), Ambion (AM4611), Thermofisher Scientific (s73188), and Ambion (AM16708) respectively. siRNA for RRM2 was acquired from Sigma Aldrich (Sense; GCGAUUUAGCCAAGAAGUUCA and antisense; UGAACUUCUUGGCUAAAUCGC). G418 (400 μg/ml), hygromycin (200 μg/ml), puromycin (1 μg/ml), or blasticidin (10 μg/ml) selection was initiated 24 hours after transfection and continued until all non-transfected cells died. Individual drug-resistant colonies were manually picked under the microscope. Individual clones and expression levels of the corresponding transgenes were validated by Western blot and/or immunostaining. For the validation of the 2C reporter ES cell line, we confirmed concomitant expression of GFP fluorescence and endogenous ZSCAN4 and MERVL-gag, together with absence of Oct3/4 expression, by immunostaining. We have stringently selected reporter lines, which fully recapitulate endogenous MERVL expression. Additionally, we performed side by side comparisons based on RNA-seq with the reporter cell lines that we and others have validated before, in Tables S1 and Table S2, see references [1,2,5-7]. We also examined Annexin-V levels in 2CLCs and ESCs, which indicated that while GFP^+ cells contain more Annexin-positive cells than GFP^- cells (5% versus 1%, respectively), GFP^+ cells are mostly Annexin-V negative (Extended Data Fig. 7j). While slight differences in the proportion of 2CLCs may vary from reporter to reporter and between experiments, fold-induction is systematically compared to controls performed in the same experiment and under identical experimental conditions. Note that, in addition to the fast maturation/folding time of tbGFP (~30 min[8]), the use of a PEST degradation signal to track 2CLCs [1,6] allows capturing 2CLCs more accurately than without PEST, in which the fluorescence also reflects 2CLC 'history' as the protein persists for longer in the cells.

Immunostaining and detection of EdU incorporation

Cells were washed with PBS, fixed for 15 min in 4% PFA in PBS at room temperature and permeabilized with 0.5% Triton X-100 in PBS for 15 min at room temperature. Cells were blocked in 5% normal goat serum in PBS for 1 h at room temperature and incubated overnight at 4°C with the primary antibodies described in Supplementary Table S7. After washing with PBS, the cells were incubated with appropriate secondary antibodies. DNA was stained with 1 µg/ml 4',6-diamidino-2-phenylindole (DAPI). For EdU incorporation analyses, cells were incubated with 50 µM EdU for 20 min for the detection of number of EdU spots and size distribution, and for 6h for the quantification of the signal intensity. Incorporated EdU was visualized by Click-iT chemistry (Life Technologies) followed by permeabilisation as described in the manufacturer's instructions. Images were acquired on a SP8 confocal laser-scanning microscope (Leica). We used STED super resolution mode for the detection of number of EdU

spots and size distribution. EdU was coupled to Alexa-594 and images were acquired with a Plan/Apo 100x NA 1.4 oil immersion STED objective (Leica) with 561 nm excitation and a pulsed 775 nm STED depletion Laser.

Analysis of EdU incorporation

For the analysis of EdU spots in 2CLCs, we set up an automated analysis pipeline in Fiji [9] and Icy[10]. First, we combined the GFP channel (indicating 2CLCs, based on the 2C::tbGFP reporter) with the EdU-channel and a manual annotation of nuclei from the DAPI signal. In Icy, EdU spots were identified with the Wavelet Spot detector[11], with Scale parameters 3, 4, and 5 active and a threshold of 200 for each Scale. We then determined which identified EdU spots lie in which nucleus region with the ROI inclusion plugin. Further, we measured the GFP intensity per nucleus region and the area of each EdU spot. For statistical analysis, we imported the results into 'R' (https://www.R-project.org), and aggregated the data to obtain EdU spot numbers per nucleus, the mean GFP intensity per nucleus, and the average spot size per nucleus. We applied an empirically derived threshold of 0.18 mean intensity units in the GFP channel as the minimum to define cells as 2-cell-like cells. Data was plotted with ggplot2 [12], statistical analysis was performed with the Wilcoxon rank-sum test in 'R'. For the quantification of signal intensity of EdU signals shown in Extended Data Fig. 2h, we used Fiji (https://imagej.net/Fiji).

Mathematical model of 2CLC emergence.

To quantitate the relationships between the transition rates of ESC into 2CLC during different cell cycle phases, we combined the results from the "steady-state" and the "block&release" experiments in a mathematical model to estimate the transition rates of ESC towards 2CLC in different cell-cycle phases. When fitting the model, rather than fixing specific values for each parameter, we only specified the ranges where the parameters can vary, thus taking into consideration their variability across conditions and experiments. Below, in the first two sections we provide a detailed description of the model, while in the third section we show how the model was fitted to the data. Briefly, our results indicate that data from the cell cycle release experiments can only be explained if the 'transition' rate (f) of ESC to 2CLC reprogramming is always higher in S-phase (f_S) as compared to either G2/M (f_{G2M}) (y axis) or G1 (f_{G1}) (x axis). The fitting in Figure 2f is represented by the gray area below the dashed line: all the values of the transition rates falling within the grey area fit the experimental data. Because the dashed line cuts the y and the x axis at values that are lower than 1 for both G2/M over S (f_{G2M}/f_S, y axis) or G1 over S (f_{G1}/f_S x axis), it follows that the transitions from ESCs to 2CLCs must occur most frequently in S-phase (e.g. else the dashed line would meet the axis at a value greater than 1).

Steady state. We modeled the dynamics of an asynchronous population of ESC with a steady-state distribution of cells across the cell-cycle phases. In the following calculations, we made these assumptions based on experimental data: (i) the rate of transition of 2CLC towards ESC as well as the rate of cell death of ESC are negligible; (ii) 2CLC do not form viable colonies and (iii) the fraction of 2CLC is much less than 1 at all times.

Under these assumptions, the following ODE system can be written:

$$\begin{cases} \dfrac{dN_E}{dt} = \varphi_E N_E(t) \\ \dfrac{dN_{2C}}{dt} = \varphi_{2C} N_{2C}(t) - \omega N_{2C}(t) + \left[\int f(\tau) P_E(\tau) d\tau\right] N_E(t) \end{cases}$$

N_E and $N_{2c}$are the numbers of ESC and 2CLC respectively, $\varphi_E$and φ_{2C} are their growth rates and ω is the cell death rate of 2CLC. $P_E(\tau)$ indicates the probability density function of ESC with an age τ and represents how the ESC are distributed across the cell-cycle phases at steady-state. $f(\tau)$ is the transition rate of ESC of age τ towards 2CLC. Note that the assumption (ii) above implies that $(\varphi_{2c} - \omega) < 0$, which will be used below in the calculation of the steady state value of N_{2C}/N_E.

While the model is able to accommodate more complex scenarios, for the sake of simplicity, here we assume that the transition rate $f(\tau)$ is constant within each phase of the cell cycle; in other words, $f(\tau)$ is a piecewise constant function:

$$f(\tau) = \begin{cases} f_{G1} \quad if \quad 0 \le \tau \le T_{G1} \\ f_s \quad if \quad T_{G1} \le \tau \le T_{G1} + T_S \\ f_{G2M} \quad if \quad T_{G1} + T_S \le \tau \le T_{G1} + T_S + T_{G2M} \end{cases}$$

where T_{G1}, T_S and T_{G2M} are the lengths of G1, S and G2/M-phase respectively and f_{G1}, f_s and f_{G2M} represent the transition rates in each of the three phases of the cell cycle.

With this $f(\tau)$, using that $(\varphi_{2c} - \omega) < 0$ (see above), we can calculate analytically the ratio between the number of 2CLC and ESC at steady-state:

$$\left(\frac{N_{2c}}{N_E}\right)_{ss} = \frac{f_{G1} P_E^{G1} + f_S P_E^S + f_{G2M} P_E^{G2M}}{\varphi_E + \omega - \varphi_{2c}}$$

where P_E^{G1}, P_E^S and P_E^{G2M} are the fractions of ESC in each cell cycle phase.

By rearranging terms, we obtain the following equation:

$$\frac{f_{G1}}{f_S}P_E^{G1} + \frac{f_{G2M}}{f_S}P_E^{G2M} = \left(\frac{N_{2c}}{N_E}\right)_{ss}\frac{\varphi_E + \omega - \varphi_{2c}}{f_S} - P_E^S$$

Block&release experiment. In the experimental design, cells are synchronised at the beginning of S-phase with a double thymidine block, then the 2CLC are removed from the culture using FACS and the number of newly emerging 2CLC is measured every hour after removing the block, for a total of 6 h (see main text and Fig. 2a, b). The previous system of ODEs can be adapted to describe the number of 2CLC during this experiment. If we consider that the vast majority of ESC are expected to be in S-phase and cell division is negligible during the time span of the experiment, we obtain that:

$$\begin{cases} N_E(t) = N_E \\ \dfrac{dN_{2C}}{dt} = \varphi_{2C}N_{2C}(t) - \omega N_{2C}(t) + f_S N_E \end{cases}$$

This gives the following solution for $N_{2c}(t)/N_E$:

$$\frac{N_{2C}(t)}{N_E} = Ae^{-(\omega-\varphi_{2C})t} + \frac{f_S}{\omega - \varphi_{2C}}$$

where A is a constant that depends on the initial conditions.

Model fitting. As we show above, the following two equations describe the fraction of 2CLC at the steady state and during the DTB block&release experiment, respectively:

$$\frac{f_{G1}}{f_S}P_E^{G1} + \frac{f_{G2M}}{f_S}P_E^{G2M} = \left(\frac{N_{2c}}{N_E}\right)_{ss}\frac{\varphi_E+\omega-\varphi_{2c}}{f_S} - P_E^S \quad (1)$$

$$\frac{N_{2C}(t)}{N_E} = Ae^{-(\omega-\varphi_{2C})t} + \frac{f_S}{\omega-\varphi_{2C}} \quad (2)$$

These equations include parameters for which we can give ranges of values based on experimental data:

- $P_E^{G1}, P_E^S, P_E^{G2M}$: the fraction of ESC in each cell cycle phase can be estimated by measuring the distribution of DNA content in a ESC population by FACS. From our data, we obtained the following estimations (averaged over 7 experiments), using respectively the Watson and the Dean-Jett-Fox (DJF) algorithms, respectively, according to the FlowJo FACS analysis software:

Watson - G1: 0.187 S: 0.573 G2M: 0.240

DJF - G1: 0.264 S: 0.401 G2M: 0.335

- $\left(\frac{N_{2c}}{N_E}\right)_{ss}$: the fraction of 2CLC at steady-state is typically between 0.2% and 1% [1,2,6]

- φ_E: the growth rate of ESC can be written as $ln(2)/T_E$, where T_E is the length of cell cycle in ESC that can vary between 8 and 10 hours [13];

- $(\omega - \varphi_{2c})$: this is the difference between the cell death rate and the growth rate of 2CLC. Note that $1/(\omega - \varphi_{2c})$ corresponds to the typical time during which 2CLC remain alive, as estimated by live-cell time-lapse microscopy, which we considered between 12 and 24 hours.

Once a particular combination of the parameters is chosen, the fitting procedure consists of two steps: during the first step, equation (2) is used to fit the data from the DTB block and release experiment to estimate f_S. We considered only measurements starting from t=3h to take into account the delay in detecting the GFP signal.

Then, in a second step, the estimation of f_S is plugged in equation (1), which describes the set of values of f_{G1}/f_S and f_{G2M}/f_S that are compatible with the data. Such values lie on a line in the $(\frac{f_{G1}}{f_S}, \frac{f_{G2M}}{f_{GS}})$ plane (see Extended Data Fig. 3f for an example of the fitting procedure and results).

While every possible combination of parameters will produce a different line in the $(\frac{f_{G1}}{f_S}, \frac{f_{G2M}}{f_S})$ plane, by varying the parameters in the ranges specified above we obtain an area that includes all possible values of f_{G1}/f_S and f_{G2M}/f_S , taking into account the experimental data as well as the variability of the parameters (see Fig. 2f)

Live-cell imaging and cell tracking

For live imaging of cell cycle dynamics and to determine the S-phase interval, we followed the procedure established by [3]. ESCs harbouring CAG::mCherry-hCdt1(1/100)Cy(-), CAG::iRFP-hGeminin(1/110), and the MERVL (2C::3xtbGFP-PEST) reporter were plated on #1.5 glass bottom dishes (MatTek corporation). Time-lapse recordings were acquired on a Nikon Ti-E microscope equipped with Bruker Opterra 2 multipoint confocal scan head, motorised stage (ASI), enclosure incubator (In Vivo Scientific), manual flow meters for air and CO_2 (Voegtlin, Germany) set to 190 ml/min and 10 ml/min, respectively, and a custom build humidifier and stage-top chamber to supply the gas mix. Images were acquired with a Plan/Apo 1.4 NA 60x oil immersion objective (Nikon) at 100 ms exposure and 70 μm slit scan mode on a Photometrics Evolve 512 Delta EMCCD Camera with EM gain set to 200. Laser power was adjusted to 10%, 100%, 100% for 488 nm, 561 nm, and 640 nm laser lines, respectively, and further attenuated with a 10% neutral density filter within the Helios Laser launch. Typically, time-lapse recordings were acquired every 15 minutes at 11 Z-planes spaced at 4 μm with

dedicated 520/40, 609/54, and 655LP emission filters (Semrock, Chroma). Images were recorded in RAW format at maximum camera transfer speed and converted to .ome.tif at the end of the acquisition. Around 15 XY positions were recorded per experiment, for up to 72 hours. Cells were tracked manually in TrackMate [14], with z-positions adjusted to the middle of the cell. Cells were traced backwards in time to identify 2CLCs after emergence of turboGFP and forward to obtain tracks of non-2CLCs. The resulting TrackMate XML file was analysed with a custom Python script to extract intensity information in each channel at each time point of the track. For the cell cycle analysis, cell track intensity data was processed in R. Cell lineages were extracted for each experiment and stage position by TrackMate cell identifiers. mCherry-hCdt1(1/100)Cy(-) and iRFP-hGeminin(1/110) intensities were normalised to minimum/maximum intensity values (see Equation below) per current cell time series, defined as either start of the time lapse until cell division, or from one cell division until the next division, or from one cell division until end of the track, or until blebbing indicated cell death, respectively. To determine S-phase length[3], we fitted a logistic model (see Equation below) to the increasing segment of the mCherry-hCdt1(1/100)Cy(-) and iRFP-hGeminin(1/110) intensities, respectively, padded by the start/end values to facilitate convergence of the non-least-square fitting algorithm[15]. The duration of S-phase was calculated as the difference of the inflection points (t0) of the curve fits. Starting values for the Levenberg-Marquardt algorithm were the maximum of the normalised intensities (*A*), the time point closest to *A*/2 (*t*0), and a fixed value of 0.0001 for k. We classified cells as 2CLC, when the GFP signal exceeded a threshold of 693.1, as determined from the average GFP background (without cells) plus three standard deviations.

Equation for minimum/maximum normalization:

$$I_{norm} = \frac{(I - I_{min})}{(I_{max} - I_{min})}$$

where I is the image intensity, I_{min} and I_{max} are minimum and maximum intensities of the current cell time series, respectively, and I_{norm} is the normalised intensity.

Equation for logistic fit:

$$y_{fit} = A\frac{1}{1 + e^{-k(t-t0)}}$$

where *A* is the curve's amplitude, k the logistic growth rate, *t* the time and *t*0 the time value of the sigmoid's infliction point.

Quantitative RT-PCR

Total RNA was extracted from ES cells with the RNeasy Plus mini kit (Qiagen) and treated with turbo DNase (Life Technologies) to remove genomic DNA. Complementary DNA was

synthesised using the ThermoScript™ RT-PCR System (Life Technologies) with random hexamers. Real-time PCR was performed with LightCycler 480 SYBR Green I Master Mix (Roche) on a LightCycler 96 Real-Time PCR System (Roche). The relative expression level of each gene was analysed with comparative Cq method and normalised to Gapdh. The primers used in this study are the following.

USP7:
Forward; 5'-GCGTGGGACTCAAAGAAGC-3'
Reverse; 5'-GAATCATCGCCCTCTGTTGG-3'
MERVL:
Forward; 5'-CTCTACCACTTGGACCATATGAC-3'
Reverse; 5'-GAGGCTCCAAACAGCATCTCTA-3'
GAPDH:
Forward; 5'-CATGGCCTTCCGTGTTCCTA-3'
Reverse; 5'-GCCTGCTTCACCACCTTCTT-3'
RRM1:
Forward; 5'-CCCAATGAGTGTCCTGGTCT-3'
Reverse; 5'-TTCTGCTGGTTGCTCTTCC-3'
RRM2:
Forward; 5'-TGCGAGGAGAATCTTCCAGGAC-3'
Reverse; 5'-CGATGGGAAAGACAACGAAGCG-3'

Western blot.

Cells were washed twice in PBS, collected and lysed on ice for 30 min in RIPA buffer with complete protease inhibitor cocktail (Roche). Following centrifugation, the supernatant was collected and the cell pellets were boiled in SDS sample buffer at 96 °C for 5 min, separated by SDS-PAGE and transferred to polyvinylidene difluoride (PVDF) membranes. The membranes were blocked in 5% skim milk and 0.1 % TWEEN 20 in PBS at room temperature for 1 hour and incubated overnight at 4 °C with the primary antibodies (See Table S7) and at room temperature for 1 hour with secondary antibody conjugated with horseradish peroxidase (HRP). HRP activity was detected by ECL western blotting detection reagent (GE healthcare) and the band intensities were quantified using Fiji.

RNA sequencing analysis and sample clustering

STAR aligner [16] was used to map sequencing reads to transcripts in the mouse mm9 reference genome. Read counts for individual transcripts were produced with HTSeq-count [17], followed by the estimation of expression values and detection of differentially expressed transcripts using EdgeR [18]. For the comparison between DE genes in endogenous and siUSP7-induced

2CLCs, log fold change of gene expression between GFP^- vs GFP^+ cells, and between siUSP7 GFP^- and siUSP7 GFP^+ cells (x and y axes in scatter plot in Extended Data Fig. 4e) was estimated using EdgeR[18]. Genes that were up-regulated, based on the cutoffs of at least 2-fold change and FDR<0.01, in both comparisons are marked red. For all analyses, differentially expressed genes were defined by at least 2-fold change with FDR less than 0.01. For the comparison between several 2CLC lines and mouse embryo stages, sample clustering was carried out as previously described[19], using gene counts obtained for each sample by STAR and HTseq. Batch effects elicited by differences across previous studies were corrected using the ComBat method implemented in the SVA package (https://bioconductor. org/packages/release/bioc/html/sva.html). A sample distance matrix was calculated using the previously described[19] correlation-based similarity method as D=1 – corr, where corr is the correlation coefficient of expression values across the gene set. The following datasets from GEO database were used: MII oocyte (GSM1933935); Zygote (GSM1625860); Early 2-cell (GSM1933937); 2-cell (GSM1625862); 4-cell (GSM1625864); 8-cell (GSM1625867); ICM (GSM1625868); 2Ctomato negative ESCs (GSM838739); 2Ctomato positive 2CLCs (GSM838738); mESC (GSM1625873); Control ES cells without treatment (E-MTAB-2684); ES cells (untreated GFP minus) (E-MTAB-2684); 2CLCs (untreated GFP plus) (E-MTAB-2684); CAF-1 KD induced 2CLCs (si-p150 GFPplus) (E-MTAB-2684); ZMYM2-depleted ESC (GSM 1933935); Dox-induced NELFA positive cells (GSM3110926); NELFA(high) (GFP positive) (GSM3110919); miR-344(DR+/+) (GSM4224405). PCA analysis was performed on log-transformed RPKM expression values across all datasets using prcomp function in R. Previously published set of genes upregulated at the 2-cell stage[20] was used as the 2-Cell transcriptional signature in the GSEA analysis[21] of gene set enrichment in the comparison between RNA-seq samples from siUSP7-2CLC and siUSP7-ESC.

Chimera Assay

Collected zygotes were grown for two days in KSOM until they reached the 4-8- cell stage. Their zona pellucida was removed by short exposure to Acid Tyrode solution and individual denuded embryos were placed each in a concave microwell, created by a smooth depression using darning needles. For the donor cell preparation, we transfected siControl, siUSP7, or treated with 50 μM HU into the 2C::tbGFP reporter ESC line stably expressing H2B-tdiRFP and ESCs and the 2CLCs of each group were sorted by FACS according to their GFP fluorescence. Approximately 10 cells were aggregated with each host embryo, and cultured for an additional two days. Chimera blastocysts were fixed for 15 min in 4% PFA in PBS and permeabilised with 0.5% Triton X-100 in PBS for 15 min at room temperature. Embryos were blocked in 5% normal goat serum in PBS for 1 h and incubated with the Alexa Fluor 647 Phalloidin (Thermofisher Scientific) for 1 h at room temperature. DNA was stained with 1 μg/ml

DAPI. To analyse the contribution of donor cells, we reconstructed blastocysts in 3D using the IMARIS software, with the help of orthogonal planes, and defined outer (TE) and inner (ICM) cells, based on phallaoidin staining, which labels cortical actin, as before[22]. The use of phalloidin for cell membrane labelling allowed us to identify 'Inside' cells as those lacking any contact with the outer surface of the embryo, whereas 'outside' cells have such contact[22,23]. We discarded cells that appeared morphologically abnormal or dead (e.g. based on DAPI and/or abnormal cytoplasm as judged by phallaoidin distribution) and cells which were not fully incorporated into the blastocysts analysed. Individual cells were quantified across 96 embryos by two independent people with double blind scoring. In Extended Data Fig. 5d, data are displayed as the percentage of cells, which upon aggregation, display inner (ICM) or outer (TE) position. For the analysis of the expression of lineage markers after single cell aggregation, we aggregated individual GFP^+ cells after siUSP7 or 50μM HU treatment of the 2C::tbGFP reporter ESC line stably expressing H2B-tdiRFP into 4-cell stage embryos using a Piezo. Embryos were then cultured until the blastocyst stage, fixed, immunostained with the Oct3/4 and Cdx2 antibodies and analysed by 3D-confocal microscopy. A total of 17 and 21 embryos were analysed for the siUSP7 and 50 μM HU treatment, respectively.

Reprogramming of MEFs to iPSC

i4F MEFs were derived from the i4F mice [24] and kindly provided by Anne Dejean (Institut Pasteur). $2.5x10^5$ i4F MEFs were seeded on 6-well plates the day before the induction of reprogramming. Cells were kept in KSR medium with 1 μg/mL doxycycline for 10 days in the presence or absence of HU treatment with indicated time windows. Colonies were stained with Alkaline Phosphatase Detection Kit (Milipore) to assess the reprogramming efficiency. Colony counting was performed in Fiji using the Trainable Weka segmentation plugin. We trained a FastRandomForest classifier with the default settings and the following features: Gaussian, Sobel, Hessian, Difference of Gaussians, Membrane projections, Variance, Mean, Minimum, Maximum, Median, Structure, Entropy, and Neighbours. We defined 4 classes, colonies, background, dust, and other (non-colony containing bright image regions, mainly from light reflecting off the plastic dish). We trained the classifier on several randomly chosen images. With a custom Fiji macro, the Weka classifier model was then applied to 25 randomly chosen, 500 pixel wide square regions per well of a 6-well plate. Candidate colony labels were binarised and binary masks extended 1 pixel to merge any fragmented colonies. We then used the Particle Analyser plugin to determine shape descriptors, specifically area and roundness of the identified objects. In 'R', we then filtered out any objects smaller than 20 square pixels and of a roundness smaller than 0.2 which we empirically determined as cutoff for wrongly classified noise or very elongated objects, respectively, the latter one originating

most often from the border of the well. Plotting and statistical analysis were done in ‘R’. We applied a generalised linear model with Poisson distribution to determine whether HU treatment and/or length of treatment lead to a significant change in colony number.

Analysis of repeat elements

The annotation of repeat elements in the mm9 reference genome was downloaded from Repeatmasker (http://www.repeatmasker.org). To estimate the enrichment of a given type of repeat elements within a given set of genomic regions, the number of element copies that overlapped with these regions was compared to the random distribution of the number of overlaps between these genomic regions and the instances of repeats randomly shuffled across the genome, based on 1000 random shuffles. The average expression value for each repeat type was then estimated as RPKM by normalizing the read count by the total size of sequencing library and total genomic length of all repeats of this type.

Histone modification profiles of genes that change the replication timing

Generation of libraries and analysis of histone modifications was done globally as described[25]. ChIP-Seq data was downloaded from previously published data by Liu, X., et al.[26] for H3K4me3 and H3K27me3 (GSE73952) and Wang, C., et al.[27] for H3K9me3 (GSE97778) for the 2-cell-embryo. Oocyte H3K4me3 is from GSE73952 (ref. 26). Likewise, ESC profiles of H3K4me3, H3K9me3, and H3K27me3 were retrieved from Marks H., et al.[28] (GSE23943). Illumina TruSeq adapters and the overrepresented sequences in FastQC were trimmed using the palindrome mode of trimmomatic v0.38 under the parameters ILLUMINACLIP:Adapters:3:30:8:1:true LEADING:10 TRAILING:10 SLIDINGWINDOW:4:15 MINLEN:10. Bowtie2 was run for aligning the trimmed reads to the mm10 mouse genome vM19 (GRCm38.p6) downloaded from GENCODE. Reads were fixed using fixmate; unmapped and multimapped reads were removed. Peak calling was carried out using the callpeak function of MACS2 v2.1.2.20181002, by setting a threshold of q=0.01. Deeptools toolkit v3.1.3, was used to compute the peak scores and plot the heatmap using the functions computeMatrix and plotHeatmap, all the panels were ordered according to the clustering of H3K4me3.

Analysis of H3.3 enrichment on MERVL

ChIP-seq datasets for 2-cell stage mouse embryos[29] were aligned using bowtie2 (version 2.3.5)[30] with the options “--local --very-sensitive-local -I 5 -X 700”. Duplicates were removed using samtools (version 1.9) [31]. Only correctly paired reads were used for subsequent analyses, without multi-mapping filtering. Reads overlapping MERVL elements (MT2_Mm, MERVL-int) were quantified for each locus using bedtools (v2.26.0) and normalized by the

sequencing depth and length of the fragment. The GTF annotation used was from the TEtranscripts [32]. Statistical tests were used against the corresponding chromatin input sample using a paired Wilcoxon test. Outliers were excluded from the figure.

CUT&RUN-qPCR

CUT&RUN was performed as described[33], with slight modifications. 2C::tbGFP reporter ES cells stably expressing SNAP-H3.3 were sorted into GFP^- and GFP^+ fraction by FACS. Each population of 20000 cells was washed with wash buffer (20 mM HEPES(pH7.5), 150 mM NaCl, 0.5mM Spermidine, proteinase inhibitor cocktail (11873580001)) and resuspended with wash buffer containing Concanavalin A beads (Polysciences). After 10 min rotation, tubes were placed on magnetic stand and the liquid was discarded. The cells bound to beads were resuspended with antibody buffer (anti-SNAP antibody, 0.05 % Triton-X, 2 mM EDTA, 20 mM HEPES(pH7.5), 150 mM NaCl, 0.5 mM Spermidine, proteinase inhibitor cocktail(11873580001)) and rotated overnight at 4 ºC. Tubes were placed on the magnetic stand and beads were washed with Triton wash buffer (0.05 % Triton-X containing wash buffer). Beads were resuspended in 50 μL Triton wash buffer containing 700 ng/mL pA-MNase (gift from S. Henikoff) and rotated at room temperature for 1 h. Tubes were placed on the magnetic stand and beads were washed with Triton wash buffer followed by Low-salt rinse buffer (20 mM HEPES, 0.5 mM Spermidine, 0.05 % Triton-X, proteinase inhibitor cocktail). Then the tubes were chilled on ice and beads were incubated with Calcium isolation buffer (wash buffer containing 2 mM $CaCl_2$) for exactly 30 min. Tubes were placed on the magnetic stand and supernatants were collected. Beads were resuspended with EGTA-STOP buffer (170 mM NaCl, 20 mM EGTA, 20 μg/mL Glycogen, 25 μg/mL RNaseA, 2 pg/mL Spike-in yeast DNA (gift from S. Henikoff)) and incubated for 30 min at 37 ºC. The supernatant was collected and the DNA fragments were purified with a spin column. Library preparation was done as described[34]. Real-time PCR was performed with LightCycler 480 SYBR Green I Master Mix (Roche) on a LightCycler 96 Real-Time PCR System (Roche). The fold changes of relative enrichment of H3.3 were analysed with comparative Cq method. The primers used in this study are the following.

MERVL:

Forward; 5'-CTCTACCACTTGGACCATATGAC-3'

Reverse; 5'-GAGGCTCCAAACAGCATCTCTA-3'

Single embryo RNA sequencing analysis

Analyses were carried out on R (version 4.0.2). Reads were aligned with STAR (2.7.3a) [16] to the mm10 genome with the default settings and counting the reads for every gene using the

option “--quantMode GeneCounts”. The index was created using the mm10 annotation from iGenomes from the UCSC source, the ERCC spike-in control genes (Thermofisher catalog #4456739), and the mitochondrial genes (ENSEMBL annotation downloaded from UCSC) were added to the index. FPKM values were calculated for each sample using the sum of all the non-ERCC counts and the number of exonic kilobases for each gene as scaling factors. We applied the following quality thresholds and kept cells with: less than 50% of ERCC counts; more than 5000 genes detected; >500000 reads mapping to genes; <10% mitochondrial reads. ERCC threshold was set to 50% to take into account the low transcriptional complexity and RNA content of cumulus cells (CC), compared to embryos. All embryo samples showed ERCC percentages lower than 10%. Based on the above QC, seven CC cells did not pass the thresholds and were removed from the analysis. For the embryonic PCA analysis, we used the processed RPKMs from available scRNA-seq data of mouse embryos[35] that were downloaded from GEO and merged with the HU-treated and control cells FPKM data. All datasets were transformed into normalized 2-based logarithmic counts and used to compute the PCA. For the ‘RRR’ analysis, the number of reads within Reprogramming Resistant Regions[36] was quantified using bedtools (v2.26.0)[37] and normalized to counts per million (CPM). Plots were produced using ggplot2 (3.3.2)[12]. For the heatmap depicting expression of ZGA genes, we used the Database of Transcriptome in Mouse Early Embryos (version 1)[38]. Genes of the cluster of the “Major ZGA” were filtered for those with a correlation greater than 0.75 and FPKM values greater than 3. The heatmap was plotted with pheatmap with a complete linkage hierarchical clustering was applied to rows and columns.

References for Supplementary Methods

1. Ishiuchi, T. *et al.* Early embryonic-like cells are induced by downregulating replication-dependent chromatin assembly. *Nature structural & molecular biology* **22**, 662-71 (2015).
2. Macfarlan, T.S. *et al.* Embryonic stem cell potency fluctuates with endogenous retrovirus activity. *Nature* **487**, 57-63 (2012).
3. Sakaue-Sawano, A. *et al.* Genetically Encoded Tools for Optical Dissection of the Mammalian Cell Cycle. *Mol Cell* **68**, 626-640 e5 (2017).
4. Sladitschek, H.L. & Neveu, P.A. MXS-Chaining: A Highly Efficient Cloning Platform for Imaging and Flow Cytometry Approaches in Mammalian Systems. *PLoS One* **10**, e0124958 (2015).
5. De Iaco, A. *et al.* DUX-family transcription factors regulate zygotic genome activation in placental mammals. *Nature genetics* **49**, 941-945 (2017).
6. Rodriguez-Terrones, D. *et al.* A molecular roadmap for the emergence of early-embryonic-like cells in culture. *Nat Genet* **50**, 106-119 (2018).
7. Hendrickson, P.G. *et al.* Conserved roles of mouse DUX and human DUX4 in activating cleavage-stage genes and MERVL/HERVL retrotransposons. *Nature genetics* **49**, 925-934 (2017).
8. Evdokimov, A.G. *et al.* Structural basis for the fast maturation of Arthropoda green fluorescent protein. *EMBO Rep* **7**, 1006-12 (2006).

9. Schindelin, J. *et al.* Fiji: an open-source platform for biological-image analysis. *Nat Methods* **9**, 676-82 (2012).
10. de Chaumont, F. *et al.* Icy: an open bioimage informatics platform for extended reproducible research. *Nat Methods* **9**, 690-6 (2012).
11. Olivo-Marin, J.C. Extraction of spots in biological images using multiscale products. . *Pattern Recognition* **35**, 1989-1996 (2002).
12. Wickham, H. ggplot2 : Elegant Graphics for Data Analysis. in *Use R!,* 2nd edn 1 online resource (XVI, 260 pages 232 illustrations, 140 illustrations in color (Springer International Publishing : Imprint: Springer,, Cham, 2016).
13. Hindley, C. & Philpott, A. The cell cycle and pluripotency. *Biochem J* **451**, 135-43 (2013).
14. Tinevez, J.Y. *et al.* TrackMate: An open and extensible platform for single-particle tracking. *Methods* **115**, 80-90 (2017).
15. Elzhov, V.T., Mullen, M.K., Spiess, A. & Bolker, B. minpack.lm: R Interface to the Levenberg-Marquardt Nonlinear Least-Squares Algorithm Found in MINPACK, Plus Support for Bounds. (2016).
16. Dobin, A. *et al.* STAR: ultrafast universal RNA-seq aligner. *Bioinformatics* **29**, 15-21 (2013).
17. Anders, S., Pyl, P.T. & Huber, W. HTSeq--a Python framework to work with high-throughput sequencing data. *Bioinformatics* **31**, 166-9 (2015).
18. Robinson, M.D., McCarthy, D.J. & Smyth, G.K. edgeR: a Bioconductor package for differential expression analysis of digital gene expression data. *Bioinformatics* **26**, 139-40 (2010).
19. Hu, Z. *et al.* Maternal factor NELFA drives a 2C-like state in mouse embryonic stem cells. *Nat Cell Biol* **22**, 175-186 (2020).
20. Wu, J. *et al.* The landscape of accessible chromatin in mammalian preimplantation embryos. *Nature* **534**, 652-7 (2016).
21. Subramanian, A. *et al.* Gene set enrichment analysis: a knowledge-based approach for interpreting genome-wide expression profiles. *Proc Natl Acad Sci U S A* **102**, 15545-50 (2005).
22. Torres-Padilla, M.E., Parfitt, D.E., Kouzarides, T. & Zernicka-Goetz, M. Histone arginine methylation regulates pluripotency in the early mouse embryo. *Nature* **445**, 214-8 (2007).
23. Dietrich, J.E. & Hiiragi, T. Stochastic patterning in the mouse pre-implantation embryo. *Development* **134**, 4219-31 (2007).
24. Abad, M. *et al.* Reprogramming in vivo produces teratomas and iPS cells with totipotency features. *Nature* **502**, 340-5 (2013).
25. Van Rechem, C. *et al.* Collective regulation of chromatin modifications predicts replication timing during cell cycle. *Cell Rep* **37**, 109799 (2021).
26. Liu, X. *et al.* Distinct features of H3K4me3 and H3K27me3 chromatin domains in pre-implantation embryos. *Nature* **537**, 558-562 (2016).
27. Wang, C. *et al.* Reprogramming of H3K9me3-dependent heterochromatin during mammalian embryo development. *Nat Cell Biol* **20**, 620-631 (2018).
28. Marks, H. *et al.* The transcriptional and epigenomic foundations of ground state pluripotency. *Cell* **149**, 590-604 (2012).
29. Ishiuchi, T. *et al.* Reprogramming of the histone H3.3 landscape in the early mouse embryo. *Nat Struct Mol Biol* **28**, 38-49 (2021).
30. Langmead, B. & Salzberg, S.L. Fast gapped-read alignment with Bowtie 2. *Nature methods* **9**, 357-9 (2012).
31. Li, H. *et al.* The Sequence Alignment/Map format and SAMtools. *Bioinformatics* **25**, 2078-9 (2009).
32. Jin, Y., Tam, O.H., Paniagua, E. & Hammell, M. TEtranscripts: a package for including transposable elements in differential expression analysis of RNA-seq datasets. *Bioinformatics* **31**, 3593-9 (2015).
33. Skene, P.J. & Henikoff, S. An efficient targeted nuclease strategy for high-resolution mapping of DNA binding sites. *Elife* **6**(2017).

34. Hainer, S.J. & Fazzio, T.G. High-Resolution Chromatin Profiling Using CUT&RUN. *Curr Protoc Mol Biol* **126**, e85 (2019).
35. Deng, Q., Ramskold, D., Reinius, B. & Sandberg, R. Single-cell RNA-seq reveals dynamic, random monoallelic gene expression in mammalian cells. *Science* **343**, 193-6 (2014).
36. Matoba, S. *et al.* Embryonic development following somatic cell nuclear transfer impeded by persisting histone methylation. *Cell* **159**, 884-95 (2014).
37. Quinlan, A.R. & Hall, I.M. BEDTools: a flexible suite of utilities for comparing genomic features. *Bioinformatics* **26**, 841-2 (2010).
38. Park, S.J., Shirahige, K., Ohsugi, M. & Nakai, K. DBTMEE: a database of transcriptome in mouse early embryos. *Nucleic Acids Res* **43**, D771-6 (2015).
