# Supplementary material for: DNA replication fork speed underlies cell fate changes and promotes reprogramming
Source: Nat Genet. 2022 Mar 7;54(3):318–27. doi: 10.1038/s41588-022-01023-0 (PMC8920892; doi:10.1038/s41588-022-01023-0)
Supplement: Supplementary file 2 — Reporting Summary. [file 41588_2022_1023_MOESM2_ESM.pdf]

nature portfolio

Corresponding author(s): Maria-Elena Torres-Padilla

Last updated by author(s): Nov 19, 2021

Reporting Summary

Nature Portfolio wishes to improve the reproducibility of the work that we publish. This form provides structure for consistency and transparency in reporting. For further information on Nature Portfolio policies, see our Editorial Policies and the Editorial Policy Checklist.

Statistics

For all statistical analyses, confirm that the following items are present in the figure legend, table legend, main text, or Methods section.

n/a	Confirmed	
☐	☒	The exact sample size (*n*) for each experimental group/condition, given as a discrete number and unit of measurement
☐	☒	A statement on whether measurements were taken from distinct samples or whether the same sample was measured repeatedly
☐	☒	The statistical test(s) used AND whether they are one- or two-sided<br>*Only common tests should be described solely by name; describe more complex techniques in the Methods section.*
☒	☐	A description of all covariates tested
☐	☒	A description of any assumptions or corrections, such as tests of normality and adjustment for multiple comparisons
☐	☒	A full description of the statistical parameters including central tendency (e.g. means) or other basic estimates (e.g. regression coefficient) AND variation (e.g. standard deviation) or associated estimates of uncertainty (e.g. confidence intervals)
☐	☒	For null hypothesis testing, the test statistic (e.g. *F*, *t*, *r*) with confidence intervals, effect sizes, degrees of freedom and *P* value noted<br>*Give P values as exact values whenever suitable.*
☒	☐	For Bayesian analysis, information on the choice of priors and Markov chain Monte Carlo settings
☒	☐	For hierarchical and complex designs, identification of the appropriate level for tests and full reporting of outcomes
☒	☐	Estimates of effect sizes (e.g. Cohen's *d*, Pearson's *r*), indicating how they were calculated

Our web collection on statistics for biologists contains articles on many of the points above.

Software and code

Policy information about availability of computer code

Data collection	LAS X (version 3) and Prairie View (version 5) were used for imaging data acquisition and basic image manipulation. BD FACSDiva software (version 8) was used for cell sorting data acquisition.
Data analysis	Microscoft excel (version 15) were used for data analysis. Adobe Illustrator CS6 (version 16) and Adobe Photoshop CS6 (version 13) were used for Figure preparation. FlowJo (version 10), BD FACSDiva software (version 8), and BD FACSChorus (version 2.0) were used for analysis of cell cycle distribution and cell population. Illumina TruSeq adapters and the overrepresented sequences in FastQC were trimmed using the palindrome mode of trimmomatic v0.38 under the parameters ILLUMINACLIP:Adapters:3:30:8:1:true LEADING:10 TRAILING:10 SLIDINGWINDOW:4:15 MINLEN:10. Bowtie2 was run for aligning the trimmed reads to the mm10 mouse genome vM19 (GRCm38.p6) downloaded from GENCODE. Reads were fixed using fixmate; unmapped and multimapped reads were removed. Peak calling was carried out using the callpeak function of MACS2 v2.1.2.20181002, by setting a threshold of q=0.01. Deeptools toolkit v3.1.3, was used to compute the peak scores and plot the heatmap using the functions computeMatrix and plotHeatmap. For RNA sequencing analysis and sample clustering, STAR aligner was used to map sequencing reads to transcripts in the mouse mm9 reference genome. Read counts for individual transcripts were produced with HTSeq-count, followed by the estimation of expression values and detection of differentially expressed transcripts using EdgeR. For Analysis of H3.3 enrichment on MERVL, reads overlapping MERVL elements (MT2_Mm, MERVL-int) were quantified for each locus using bedtools (v2.26.0) and normalized by the sequencing depth and length of the fragment. The GTF annotation used was from the TEtranscripts. For Single embryo RNA sequencing analysis, analyses were carried out on R (version 4.0.2). Reads were aligned with STAR (2.7.3a) to the mm10 genome with the default settings and counting the reads for every gene using the option "--quantMode GeneCounts".

For manuscripts utilizing custom algorithms or software that are central to the research but not yet described in published literature, software must be made available to editors and reviewers. We strongly encourage code deposition in a community repository (e.g. GitHub). See the Nature Portfolio guidelines for submitting code & software for further information.

Data

Policy information about availability of data

All manuscripts must include a data availability statement. This statement should provide the following information, where applicable:

- Accession codes, unique identifiers, or web links for publicly available datasets
- A description of any restrictions on data availability
- For clinical datasets or third party data, please ensure that the statement adheres to our policy

Sequencing data generated during this study have been deposited in the Gene Expression Omnibus (GEO) under accession codes GSE136228 (Repli-seq and RNA-seq data).
Previously published RNA-seq datasets re-analysed here are available under accession codes GSM1933935 (MII oocyte); GSM1625860 (Zygote); GSM1933937 (Early 2-cell); GSM1625862 (2-cell); GSM1625864 (4-cell); GSM1625867 (8-cell); GSM1625868 (ICM); GSM838739 (2Ctomato negative ESCs); GSM838738 (2Ctomato positive 2CLCs); GSM1625873 (mESC); E-MTAB-2684 (Control ES cells without treatment); E-MTAB-2684 (ES cells, untreated GFP minus); E-MTAB-2684 (2CLCs, untreated GFP plus); E-MTAB-2684 (CAF-1 KD induced 2CLCs, si-p150 GFPplus); GSM 1933935 (ZMYM2-depleted ESC); GSM3110926 (Dox-induced NELFA positive cells) ; GSM3110919 (NELFA(high) GFP positive); GSM4224405 (miR-344(DR+/+)).
Previously published ChIP-seq datasets re-analysed here are available under accession codes GSE73952 (H3K4me3 and H3K27me3, 2-cell-embryo); GSE97778 (H3K9me3, 2-cell-embryo); GSE23943 (H3K4me3, H3K9me3, and H3K27me3, ESC); GSE74952 (H3K4me3, oocyte); GSE139527 (H3.3, 2-cell-embryo).
Figures with associated raw data are as follows: Fig. 3e, 3f, 4a-k, 5b, 5c, Extended Data Fig. 4d-g, 6b-l, 7b-f, 7h, and 7i.

Field-specific reporting

Please select the one below that is the best fit for your research. If you are not sure, read the appropriate sections before making your selection.

☒ Life sciences ☐ Behavioural & social sciences ☐ Ecological, evolutionary & environmental sciences

For a reference copy of the document with all sections, see nature.com/documents/nr-reporting-summary-flat.pdf

Life sciences study design

All studies must disclose on these points even when the disclosure is negative.

Sample size	To do the statistical test, at least 3 biological replicates were included (unless otherwise stated) based on previously published work and preliminary studies as standard for this field of research. See Figures legends for each experiment.
Data exclusions	No data were excluded.
Replication	All attempts at replication were successful as reported in the manuscript
Randomization	Cells were allocated at random to experimental groups as stated in the Methods
Blinding	Double-blind counting was carried out for Extended Data Fig. 5d in which relatively subjective counting was performed. All other analysis was objectively performed using automated approaches.

Reporting for specific materials, systems and methods

We require information from authors about some types of materials, experimental systems and methods used in many studies. Here, indicate whether each material, system or method listed is relevant to your study. If you are not sure if a list item applies to your research, read the appropriate section before selecting a response.

Materials & experimental systems

n/a	Involved in the study	
☐	☒	Antibodies
☐	☒	Eukaryotic cell lines
☒	☐	Palaeontology and archaeology
☐	☒	Animals and other organisms
☒	☐	Human research participants
☒	☐	Clinical data
☒	☐	Dual use research of concern

Methods

n/a	Involved in the study	
☒	☐	ChIP-seq
☐	☒	Flow cytometry
☒	☐	MRI-based neuroimaging

Antibodies

Antibodies used	Antibodies used were as follows: anti-BrdU(IdU) (BD Biosciences 347580), anti-BrdU(CldU) (Novus NB500-169, Abcam ab6326), anti-

Antibodies used	single stranded DNA (Milipore MAB3034), anti-Zscan4 (Milipore AB3430), anti-Oct3/4 (BD Biosciences 611203, MBL PM048), anti-Cdx2 (BioGenex AM392-5M), anti-Usp7 (BETHYL A300-034), anti-Gapdh (Milipore MAB374), anti-BrdU (Sigma B8434), anti-gH2AX (Abcam ab22551), anti-SNAP (NEB P9310), Secondary antibodies used were A11001, A121429, A11077, A21236, A16078, and A16110. More detailed information of antibodies are written in Supplementary Table S7.
Validation	Manufacturer validated that three anti-BrdU (BD Biosciences 347580), (Novus NB500-169), and (Abcam ab6326) recognize IdU (https://www.bdbiosciences.com/us/applications/research/apoptosis/purified-antibodies/purified-mouse-anti-brdu-b44/p/347580), CldU (https://www.novusbio.com/products/bromodeoxyuridine-brdu-antibody-bu1-75-icr1-_nb500-169), and CldU(https://www.abcam.com/brdu-antibody-bu175-icr1-proliferation-marker-ab6326.html), respectively. Anti-BrdU (Sigma B8434) were reported to be applicanle for IP (Shibata, Elife, 2016). Anti-single stranded DNA (Milipore MAB3034) was validated by the manufacturer (https://www.merckmillipore.com/DE/de/product/Anti-DNA-Antibody-single-stranded-clone-16-19,MM_NF-MAB3034?ReferrerURL=https%3A%2F%2Fwww.google.com%2F&bd=1). Anti-Usp7 (BETHYL A300-034) was validated by Western blot combination with siRNA experiment. Anti-Zscan4 (Milipore AB3430), anti-Oct3/4 (BD Biosciences 611203), and anti-Cdx2 (BioGenex AM392-5M) were validated our previous studies (Ishiuchi, NSCB, 2015, Rodriguez-Terrones, Nat. Genet., 2018, Burton, NCB, 2020). Anti-gH2AX (https://www.abcam.com/gamma-h2ax-phospho-s139-antibody-3f2-ab22551.html), anti-Oct3/4 (https://ruo.mbl.co.jp/bio/dtl/A/?pcd=PM048), and anti-SNAP (https://international.neb.com/products/p9310-anti-snap-tag-antibody-polyclonal#Product%20Information) were validated by manufacturerer.

Eukaryotic cell lines

Policy information about cell lines

Cell line source(s)	All of ES cells originating from our previous study (Miyanari, Nature, 2012 and Ishiuchi, NSCB, 2015).
Authentication	We have validated that our 2C-reporter cell line reflect endogenous expression of MERV-L by IF and also performed side by side comparisons based on RNAseq with the reporter cell lines that we and others have validated before (Ishiuchi, NSCB, 2015, Macfarlan, Nature, 2012, De Iaco, Nat. genet., 2017, Hendrickson, Nat. genet., 2017, Rodriguez-Terrones, Nat. Genet., 2018). Validation of FUCCI cells was done by sorting mCherry-hCdt1(1/100)Cy(-)-positive, iRFP-hGeminin(1/110)-negative cells and confirmed that subpopulation corresponded to G1 peak which was obtained by PI staining. Knock-in of the AID cassette was validated by genomic PCR with specific primer sets after the drug selection and homoallelic mutant were used for the experiments.
Mycoplasma contamination	All cell lines tested negative for mycoplasma contamination.
Commonly misidentified lines (See ICLAC register)	No commercially misidentified cell lines were used.

Animals and other organisms

Policy information about studies involving animals; ARRIVE guidelines recommended for reporting animal research

Laboratory animals	F1 (C57BL6 X CBA/H) mice were used to provide oocytes and crossed with F1 males to provide zygotes.
Wild animals	This study did not use wild animals.
Field-collected samples	This study did not involve field-collected samples.
Ethics oversight	All experiments were performed under the authorization of French legislation or the Upper Bavarian authorities.

Note that full information on the approval of the study protocol must also be provided in the manuscript.

Flow Cytometry

Plots

Confirm that:

☒ The axis labels state the marker and fluorochrome used (e.g. CD4-FITC).

☒ The axis scales are clearly visible. Include numbers along axes only for bottom left plot of group (a 'group' is an analysis of identical markers).

☒ All plots are contour plots with outliers or pseudocolor plots.

☒ A numerical value for number of cells or percentage (with statistics) is provided.

Methodology

Sample preparation	For isolation and quantification of 2CLCs, cells were washed with twice with PBS and treated with 0.25% trypsin. After neutralization with ESC medium, cells were collected by centrifugation and the dissociated single cells were resuspended in ESC medium. To calculate the population of 2CLCs, we counted turboGFP-positive ESCs after extrusion of dead and doublet cells based on the forward and side scatter profiles. After sorting, cells were collected in normal culture medium and kept at 4°C. For collection of cells in G1-phase in Fig 2e and Extended Data Fig. 2e, we sorted mCherry-hCdt1(1/100)Cy(-)-positive, iRFP-hGeminin(1/110)-negative subpopulation based on their fluorescence. For cell cycle analysis, the dissociated single cells

	were fixed with 70 % ethanol for 30 min. After treatment with 250 μg/mL RNaseA (Thermofisher Scientific) for 5 min, cells were treated with 50 μg/mL propidium iodide (PI) to stain DNA.
Instrument	Sorting was performed on a BD Biosciences FACSAria III.
Software	FlowJo (version 10) and BD FACSDiva software (version 8) were used for analysis of cell cycle distribution and cell population, respectively.
Cell population abundance	Whenever cell numbers were not an issue, fluorescence was verified after sorting and was usually 95-100%. Downstream experiments always confirmed a very high degree of sorting purity.
Gating strategy	Stringent gatings were always used, leaving a significant gap in between negative/positive or low/high populations.

☒ Tick this box to confirm that a figure exemplifying the gating strategy is provided in the Supplementary Information.
