# Supplementary figures and images for: DNA replication fork speed underlies cell fate changes and promotes reprogramming
Source: Nat Genet. 2022 Mar 7;54(3):318–27. doi: 10.1038/s41588-022-01023-0 (PMC8920892; doi:10.1038/s41588-022-01023-0)

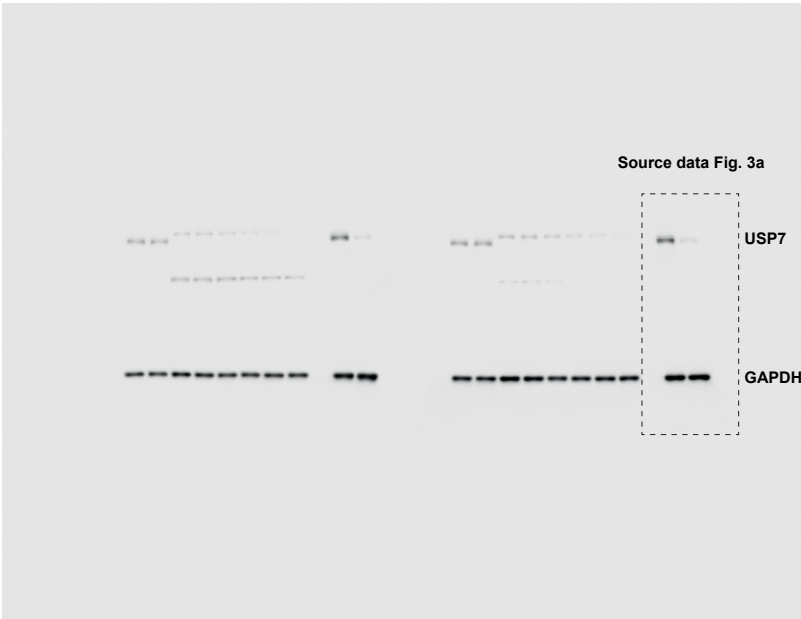

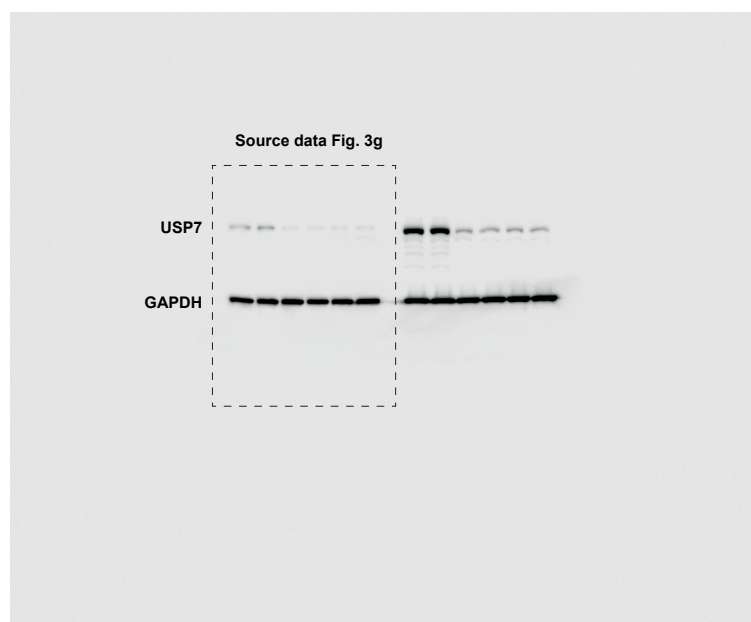

Supplement: Source Data Fig. 3 — Unprocessed western blots. [file 41588_2022_1023_MOESM4_ESM.pdf]

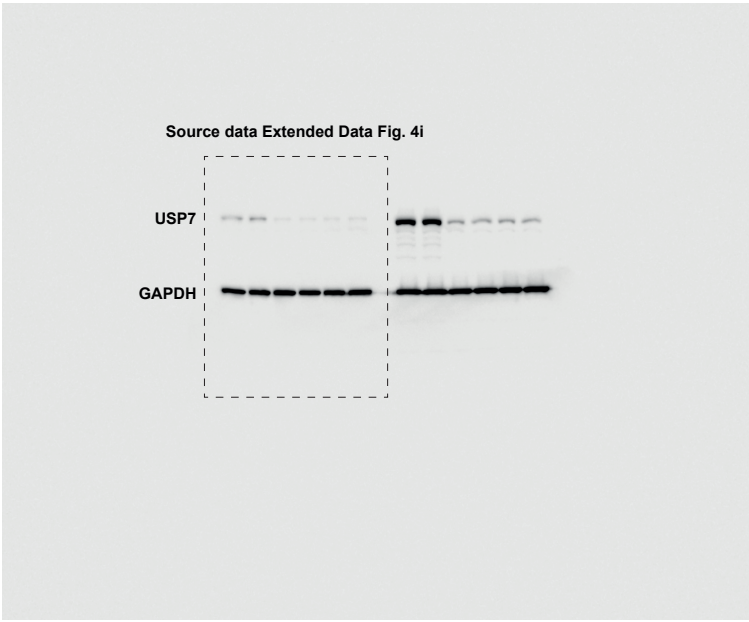

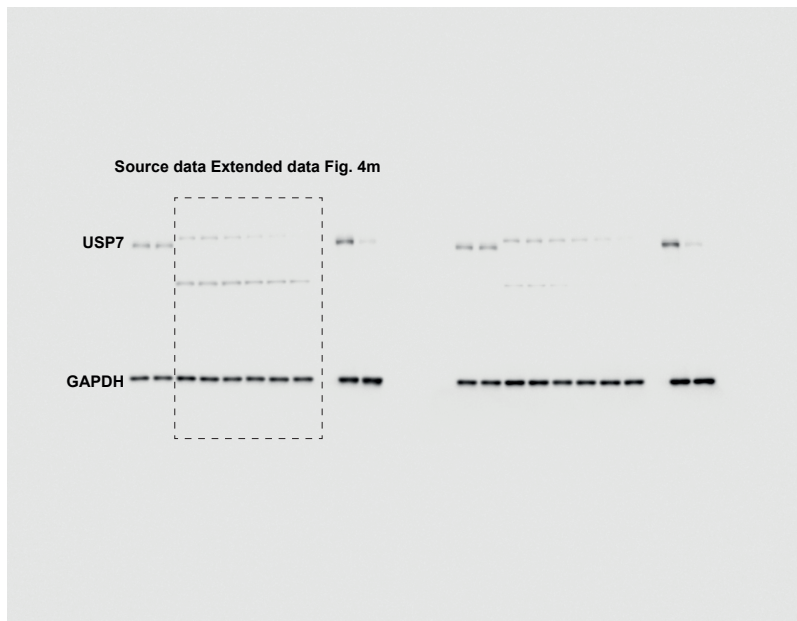

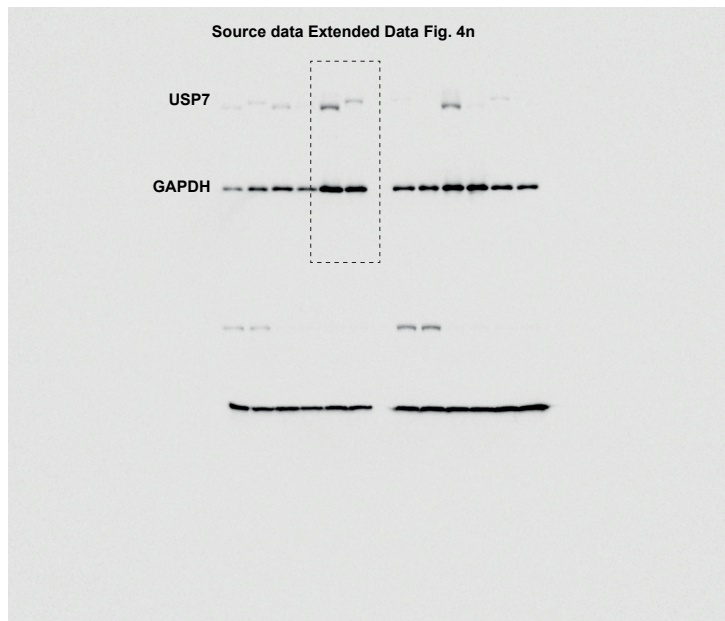

Supplement: Source Data Fig. 4 — Unprocessed western blots. [file 41588_2022_1023_MOESM5_ESM.pdf]
